# *In silico* investigation of a *KCNQ1* mutation associated with short QT syndrome

**DOI:** 10.1038/s41598-017-08367-2

**Published:** 2017-08-16

**Authors:** Ismail Adeniran, Dominic G. Whittaker, Aziza El Harchi, Jules C. Hancox, Henggui Zhang

**Affiliations:** 1 Biological Physics Group, School of Physics & Astronomy, The University of Manchester, Manchester, M13 9PL UK; 2 School of Physiology, Pharmacology and Neuroscience, Biomedical Sciences Building, University Walk, Bristol, BS8 1TD UK; 3 School of Computer Sciences and Technology, Harbin Institute of Technology, Harbin, China; 4 Space Institute of Southern China, Shenzhen, China

## Abstract

Short QT syndrome (SQTS) is a rare condition characterized by abnormally ‘short’ QT intervals on the ECG and increased susceptibility to cardiac arrhythmias and sudden death. This simulation study investigated arrhythmia dynamics in multi-scale human ventricle models associated with the SQT2-related V307L KCNQ1 ‘gain-of-function’ mutation, which increases slow-delayed rectifier potassium current (I_Ks_). A Markov chain (MC) model recapitulating wild type (WT) and V307L mutant I_Ks_ kinetics was incorporated into a model of the human ventricular action potential (AP) for investigation of QT interval changes and arrhythmia substrates. In addition, the degree of simulated I_Ks_ inhibition necessary to normalize the QT interval and terminate re-entry in SQT2 conditions was quantified. The developed MC model accurately reproduced AP shortening and reduced effective refractory period associated with altered I_Ks_ kinetics in homozygous (V307L) and heterozygous (WT-V307L) mutation conditions, which increased the lifespan and dominant frequency of re-entry in 3D human ventricle models. I_Ks_ reductions of 58% and 65% were sufficient to terminate re-entry in WT-V307L and V307L conditions, respectively. This study further substantiates a causal link between the V307L KCNQ1 mutation and pro-arrhythmia in human ventricles, and establishes partial inhibition of I_Ks_ as a potential anti-arrhythmic strategy in SQT2.

## Introduction

The short QT syndrome (SQTS) was first published as a distinct clinical entity in 2000^1^. It is characterised by an abnormally short QT interval on the electrocardiogram (ECG), tall and peaked T-waves, poor rate adaptation of the QT interval, shortened atrial and ventricular refractory periods, increased risk of atrial and ventricular arrhythmias, and an increased incidence of sudden death in affected patients^2–7^.

To date, six genetic variants of the SQTS have been identified (SQT1−6) with the first three variants (SQT1−3) being caused by gain-of-function mutations to genes encoding different K^+^ channel subunits while SQT4−SQT6 are due to loss-of-function mutations to genes encoding subunits responsible for L-type calcium channel current (I_CaL_), resulting in a mixed SQT-Brugada phenotype^8, 9^. SQT2 is caused by mutations to *KCNQ1 (KvLQT1)* ^10–13^, which in combination with *KCNE1* encodes the proteins responsible for human cardiac I_Ks_ channel^14, 15^.

The first-identified adult SQT2 variant involves a G → C substitution in nucleotide 919 of *KCNQ1* that leads to an amino acid substitution of valine to leucine on residue 307 (V307L) in the KCNQ1 channel pore helix^10^. The V307L mutation shifts the voltage dependence of KCNQ1+KCNE1 activation towards more negative voltages and accelerates channel activation, which consequently leads to increased current during ventricular repolarisation^10^. For the V307L KCNQ1 SQT2 mutation, initial *in silico* data^10^ were able to reproduce action potential (AP) shortening but did not address directly effects on the QT interval or arrhythmia mechanisms. In a subsequent study^16^, we developed a Hodgkin-Huxley (HH) style model for the V307L KCNQ1 mutation and investigated its effects in idealised one-dimensional (1D) and two-dimensional (2D) tissue. However, that study^16^ had some intrinsic limitations: (i) the developed HH model of SQT2 did not incorporate slow deactivation of the I_Ks_ with the V307L KCNQ1 mutation that was subsequently identified^17^; (ii) it did not consider possible functional consequences of realistic ventricular anatomical geometry in two and three dimensions, which play important roles in initiation and maintenance of arrhythmic excitation waves^18, 19^.

One aim of this study was to address the above limitations of the previous study using a HH model of SQT2^16^. We conducted the present study in order to: (i) develop a novel biophysically-accurate and validated Markov chain (MC) model to recapitulate the kinetic changes to I_Ks_ in the SQT2 V307L KCNQ1 mutation based on available experimental data at physiological temperature; (ii) determine the functional consequences of the SQT2 V307L mutation on AP repolarisation and the QT interval by incorporating it into a well-established human ventricular cell model^20^; (iii) explore the arrhythmogenic substrate in the SQT2 V307L mutation by using “realistic” 2D tissue and 3D organ-scale simulations; and, moreover; (iv) investigate theoretically the degree of I_Ks_ inhibition required to normalise the QT interval as a pseudo-pharmacological therapeutic intervention.

The multi-scale cardiac modelling approach taken in this study has been employed successfully in our previous studies to dissect ionic mechanisms underlying QT interval shortening and pro-arrhythmia in variants 1 and 3 of the SQTS^18, 19^. The developed MC model represents a significant advance over previous studies^10, 16^ in SQT2 modelling and the reported results further understanding of the mechanisms by which the SQT2 V307L mutation enhances susceptibility to reentrant arrhythmia. Furthermore, a theoretical basis for pharmacological intervention in SQT2 is proposed.

## Results

### Simulation of single cell I_Ks_ under control and V307L mutation conditions

**Figure 1:**
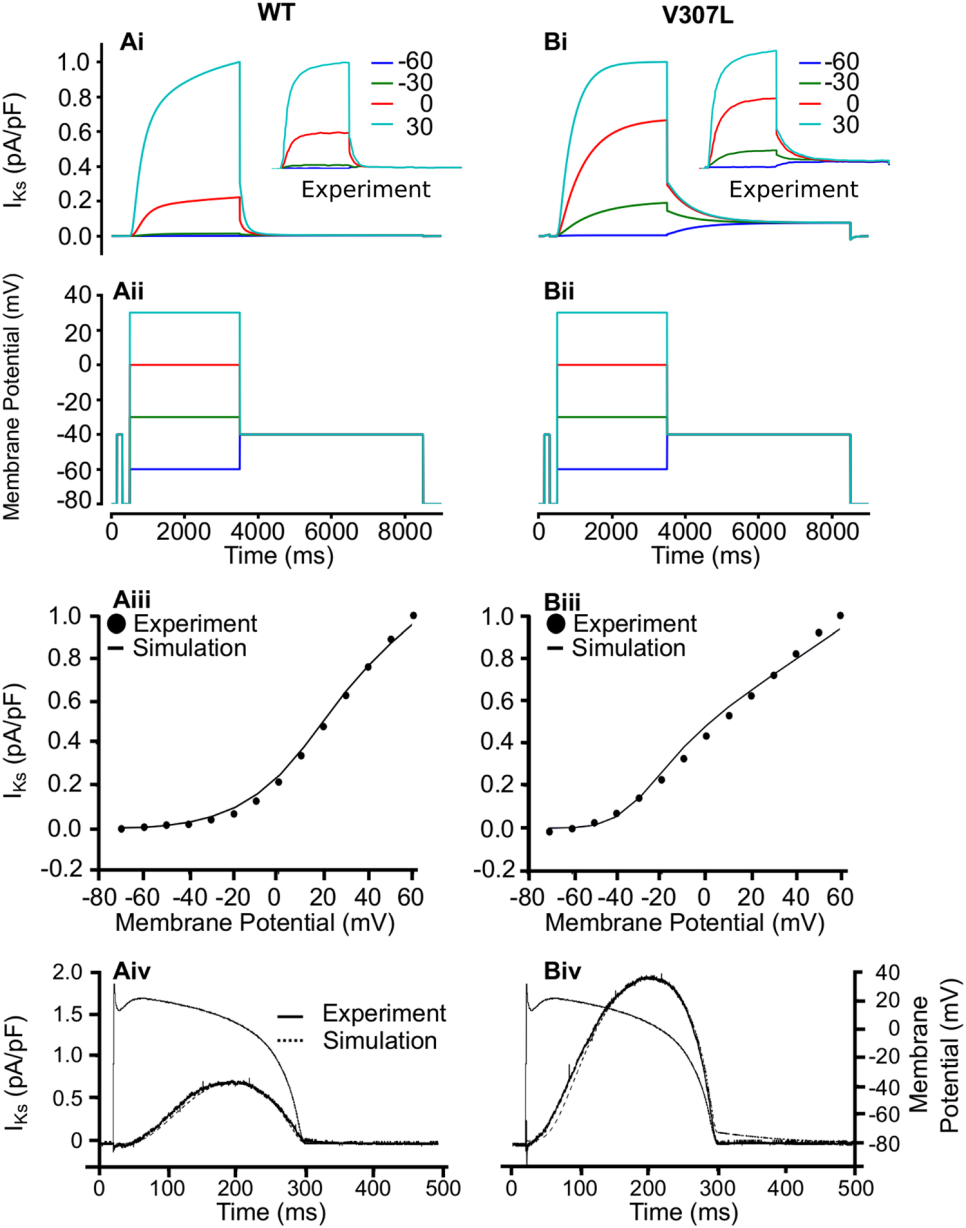
Simulated voltage and AP clamp experiments for I_Ks_. Current traces for WT (**Ai**) and V307L (**Bi**) KCNQ1 I_Ks_ at step potentials of −60 mV (blue), −30 mV (green), 0 mV (red) and 30 mV (cyan) using the voltage clamp protocol shown in (**Aii,Bii**). Comparison of simulated and experimental I-V relationship for I_Ks_ under WT (**Aiii**) and V307L (**Biii**) conditions. I_Ks_ profile during AP clamp using a ventricular AP waveform voltage command in WT (**Aiv**) and V307L (**Biv**) conditions. All experimental data, including insets shown in panels **Ai** and **Bi**, are taken from El Harchi *et al*.^17^.

First, we tested the ability of the I_Ks_ MC model to reproduce the previously published experimental data^17^ on the voltage dependence of wild type (WT) and V307L KCNQ1+KCNE1 I_Ks_ at physiological temperature. Figures 1Aii and 1Bii show the voltage clamp protocol used which is the same as that used for the experimental data^17^ and the generated I_Ks_ current traces (Fig. 1Ai and Bi) from which the I-V relationships were reconstructed (Fig. 1Aiii and Biii). The simulated I-V relationships for both the WT and *KCNQ1* V307L conditions match those recorded experimentally^17^. The simulated current traces match experimental recordings; under the V307L mutation condition, the slower deactivation rate and faster activation rate of the I_Ks_ channel^17^ compared to the WT condition were reproduced (Fig. 1Ai and Bi).

To validate the MC model formulations, we next simulated AP voltage clamps to test their ability to reproduce the dynamic properties of WT and V307L KCNQ1 mutant I_Ks_ channels. Figure 1Aiv and Biv show the results of the simulated AP clamp experiments compared to those obtained *in vitro* ^17^. The MC model reproduced the profile of I_Ks_ during imposed AP commands with a high degree of accuracy, both in the WT condition and with augmented I_Ks_ in simulations incorporating the *KCNQ1* V307L mutation.

**Figure 2:**
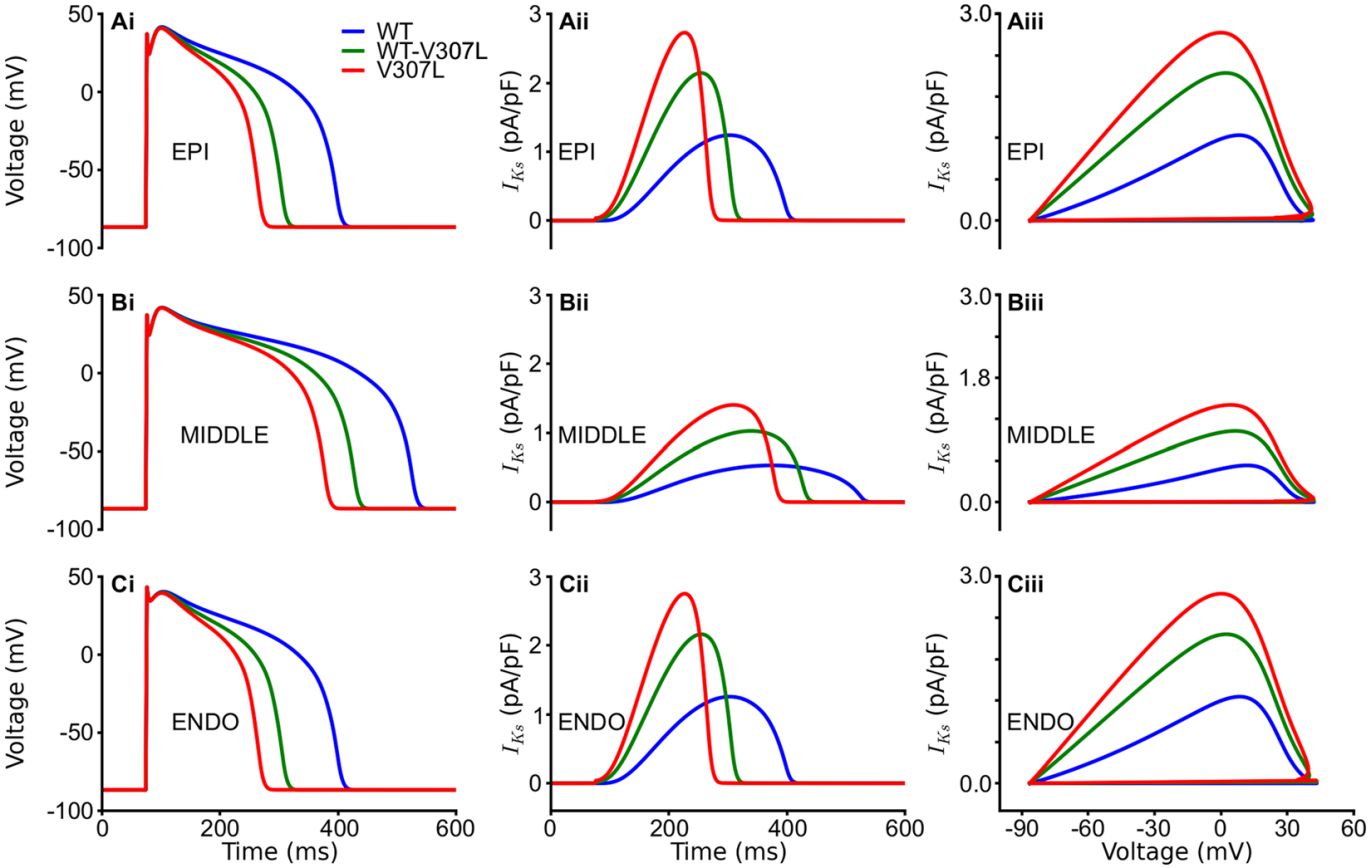
Action potentials and I_Ks_ profiles. Steady state APs (i) for EPI (**A**), MIDDLE (**B**), and ENDO (**C**) cells in WT (blue), WT-V307L (green), and V307L (red) conditions at a pacing rate of 1 Hz. Corresponding I_Ks_ profiles (ii) and I-V relationships (iii) for EPI (**A**), MIDDLE (**B**), and ENDO (**C**) cells.

Following incorporation of the WT and V307L MC models into the 2006 model of ten Tusscher *et al*. (TNNP)^20^, Fig. 2 shows simulated APs (Ai), I_Ks_ profile (Aii) and I_Ks_ instantaneous I-V relationship for an epicardial (EPI) cell (Aiii). The mid-myocardial (MIDDLE) and endocardial (ENDO) counterparts are shown in Fig. 2B and C, respectively. WT I_Ks_ increased progressively following the upstroke of the AP and reached maximal amplitude very late during the plateau phase before declining rapidly during terminal repolarisation. WT-V307L I_Ks_ activated earlier than WT and increased in amplitude more rapidly. Compared to WT current, it reached significantly higher maximal amplitude early during the plateau leading to abbreviation of the AP duration (APD). V307L I_Ks_ activated even earlier than WT-V307L I_Ks_, increased even more rapidly, and attained higher maximal amplitude. Consequently, it abbreviated the APD to a greater extent. Under the WT condition, the computed APD_90_ was 326 ms, 454 ms and 327 ms for EPI, MIDDLE and ENDO cells respectively. These were shortened respectively to 233 ms, 355 ms and 234 ms under the WT-V307L condition, and to 194 ms, 306 ms and 194 ms under the V307L mutation condition. The APD_90_ values for all the conditions are summarised in Table 1. The APD shortening resulted from the augmented I_Ks_ early during the plateau phase of the AP as shown by the time course of I_Ks_ (Fig. 2Aii–Cii) and the I-V phase plots in Fig. 2Aiii–Ciii. The greatest APD shortening was observed for the MIDDLE cell model (Table 1).

**Table 1:** Action potential duration in WT, WT-V307L and V307L conditions.

	**WT (ms)**	**WT-V307L (ms)**	**V307L (ms)**
EPI	325.6	233.0	193.6
		ΔAPD 92.6	ΔAPD 132.0
MIDDLE	453.6	355.4	305.9
		ΔAPD 98.2	ΔAPD 147.7
ENDO	327.4	233.8	194.0
		ΔAPD 93.6	ΔAPD 133.3

Action potential duration (APD) was measured in all transmural cell types; epicardial (EPI), mid-myocardial (MIDDLE), and endocardial (ENDO) at a frequency of 1 Hz. ΔAPD is measured relative to WT.

**Figure 3:**
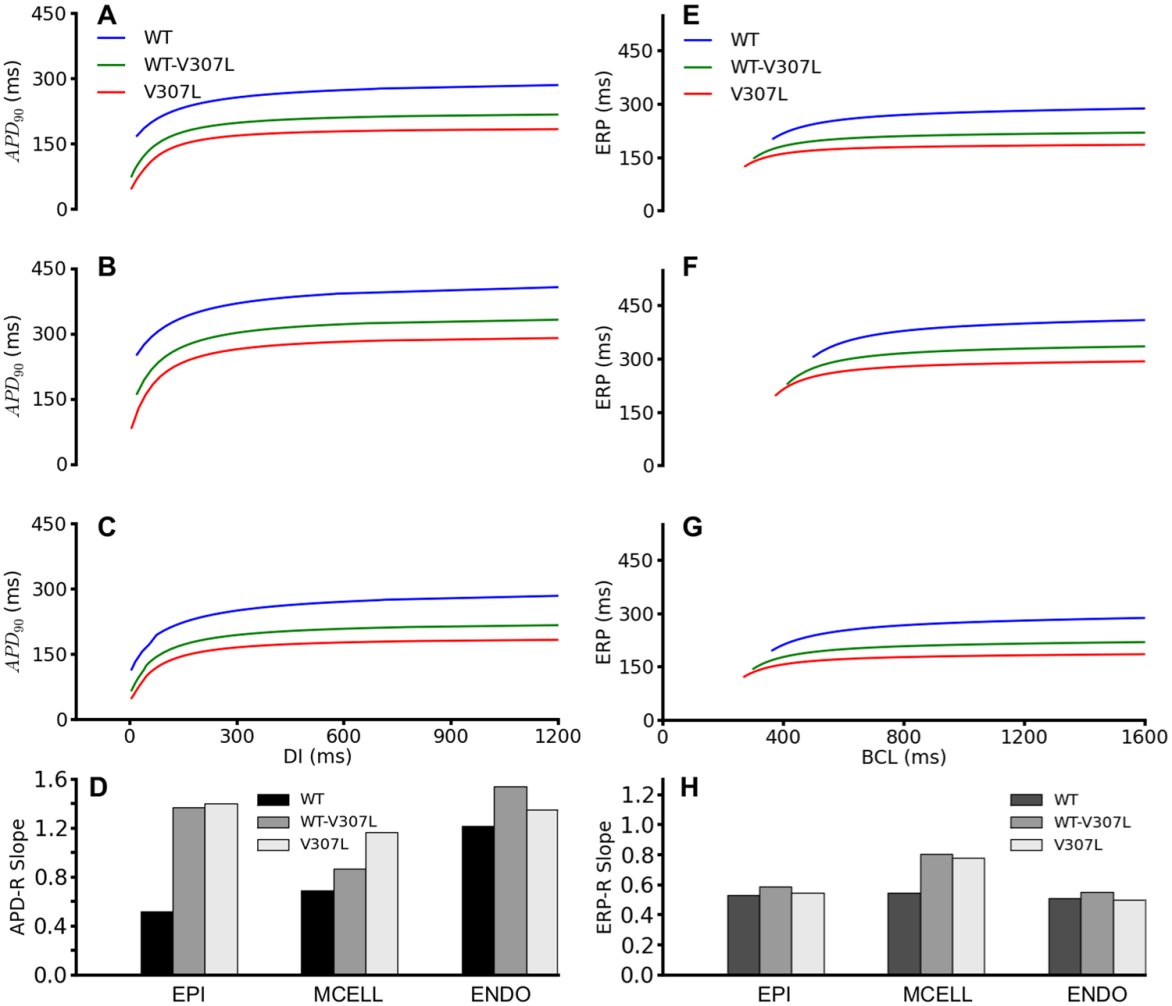
Restitution curves for APD and ERP. APD_90_ restitution curves for EPI (**A**), MIDDLE (**B**), and ENDO (**C**) cells in WT (red), WT-V307L (green), and V307L (red) conditions, with corresponding maximal slope of APD restitution (**D**). ERP restitution curves for EPI (**E**), MIDDLE (**F**), and ENDO (**G**) cells in WT (red), WT-V307L (green), and V307L (red) conditions, with corresponding maximal slope of ERP restitution (**H**).

The APD abbreviation was rate-dependent as shown by the APD restitution (APD-R) curves in Fig. 3A–C for the EPI, MIDDLE and ENDO cell types, respectively. Over the range of diastolic intervals (DI) studied, the APD was smaller in the WT-V307L and V307L mutant conditions than in the WT condition. The mutations also steepened the APD-R curves in each cell type as shown by the computed maximal slopes for each APD-R curve in Fig. 3D. In the EPI cell, the maximal slopes of the WT-V307L and V307L conditions were similar, while there was a progressive increase in steepness of the slopes in the MIDDLE cell type. In the ENDO cell, while the slopes under the WT-V307L and V307L conditions were steeper than WT, the slope of the WT-V307L condition was steeper than that of the V307L mutant.

The ERP reduction was also rate-dependent. It was reduced under the WT-V307L and V307L mutation conditions compared to the WT condition across the range of basic stimulus cycle lengths (BCL) as shown in ERP-R curves in Fig. 3E–G for the EPI, MIDDLE and ENDO cell types, respectively. In the EPI and ENDO cells, there was little difference in the slopes of the ERP-R curves between the WT, WT-V307L and V307L conditions (Fig. 3H). The slope was steeper for the mutation conditions in the MIDDLE cell compared to WT but the slope for the WT-V307L condition was slightly steeper than that for the V307L mutant alone. The mutations also shifted the ERP-R curve leftwards implying that the *KCNQ1* V307L mutation enabled ventricular cells to support higher rate electrical activity (as normally seen during VT and VF conditions).

### Simulation of the ECG with WT and SQT2 mutant I_Ks_

**Figure 4:**
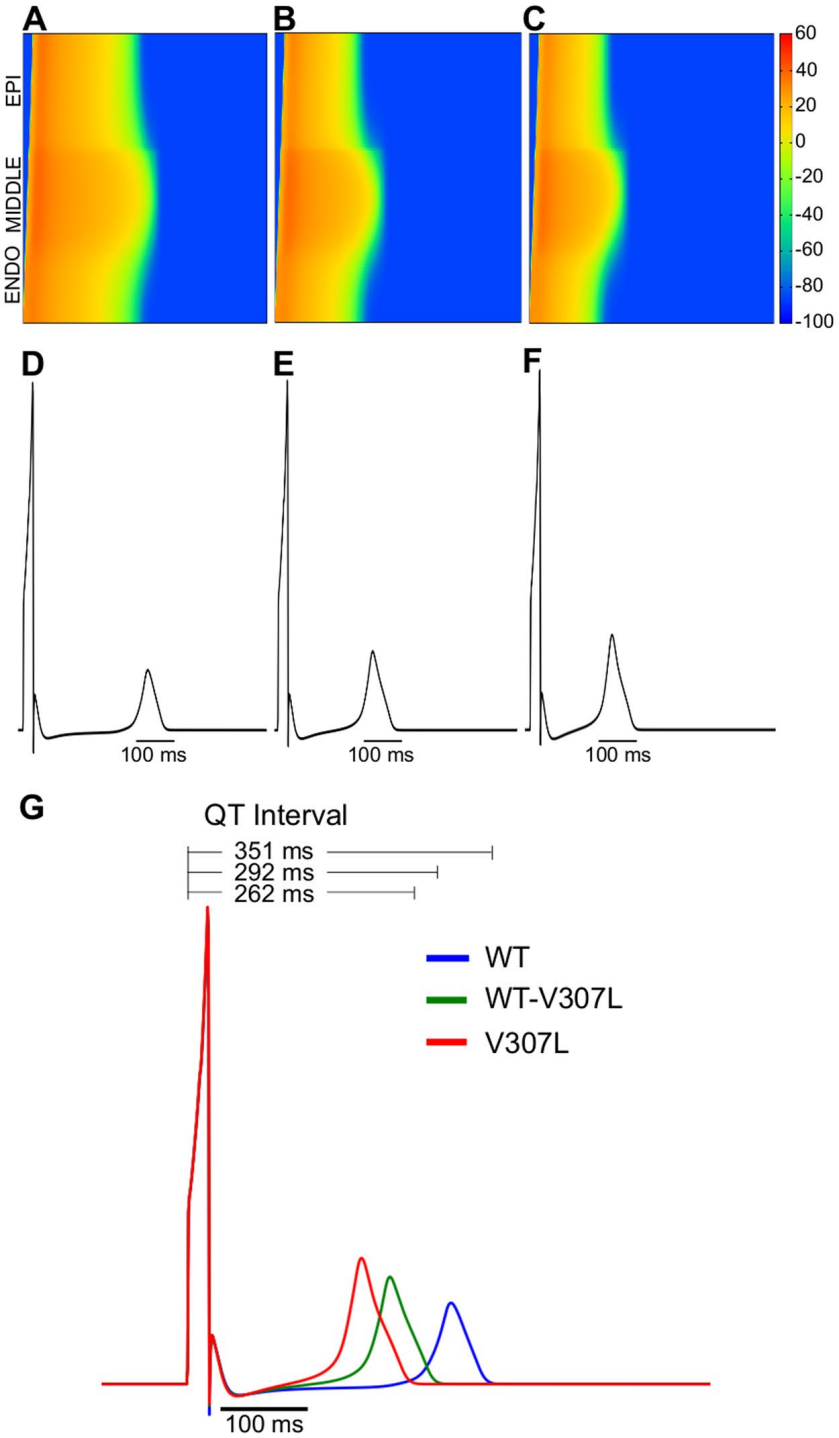
Pseudo-ECG measured in 1D strand. Space-time plot of AP propagation in a 1D transmural strand in WT (**A**), WT-V307L (**B**), and V307L (**C**) conditions, with membrane potential colour mapped from blue (−100 mV) to red (+60 mV). Space runs vertically from the endocardial (ENDO) end (bottom) to epicardial (EPI) end of the strand (top), and time runs horizontally. Pseudo-ECGs measured in WT (**D**), WT-V307L (**E**), and V307L (**F**) conditions. Superimposed pseudo-ECGs for the WT, WT-V307L, and V307L conditions, with associated QT intervals (**G**).

Using a 1D strand model of the ventricular wall, we computed a pseudo-ECG under the WT, WT-V307L and V307L KCNQ1 conditions (Fig. 4D–F). These were extracted from a propagating wave from the ENDO end of the strand towards the EPI end (Fig. 4A–C). Time runs horizontally from left to right in Fig. 4A–C while space runs vertically from the ENDO end at the bottom to the EPI end at the top. The QT interval was shortened from 351 ms in the WT condition to 292 ms in the WT-V307L condition and to 262 ms in the V307L condition (Fig. 4G). T wave width (measured as the time interval between T_peak_ and T_end_) also changed from 49 ms (WT) to 60 ms (WT-V307L) and 64 ms (V307L). These simulations thus reproduce the key features observed in the ECGs of SQTS patients; abbreviated QT interval, tall and peaked T-waves and wider T_peak_ to T_end_^2, 4–7^. As the only difference between these simulations is the altered kinetics of I_Ks_ resulting from the mutation, the observed changes in the QT interval, T wave height and width can be confidently attributed to the parameters corresponding to the V307L mutation.

Gima and Rudy^21^ showed that increased spatial gradient of membrane potential was responsible for the increase in T-wave amplitude during simulated hyperkalaemia. In order to determine if the same effect was responsible for the taller T wave amplitudes in the WT-V307L and V307L ECGs (Fig. 4D–G), we examined the effects of this KCNQ1 mutation on membrane potential heterogeneity (δV) in supplementary simulations. Supplementary Figure S2 shows the pairwise differences between EPI, MIDDLE and ENDO cells during an AP. Under the KCNQ1 WT-V307L and V307L mutation conditions, the maximal δV between EPI-MIDDLE and ENDO-MIDDLE cells were greater than under the WT condition, which contributed to the augmented T-wave amplitude^16, 18, 21^. Also shown is the spatial distribution of APD_90_, the spatial gradient of APD_90_, and the absolute value of the spatial gradient of APD_90_. The spatial gradient of APD_90_ was augmented across the strand but markedly so in the ENDO region.

### Investigating the arrhythmogenic substrate in SQT2 – 1D simulations

In further supplementary simulations using the 1D strand, we investigated the vulnerability of WT, WT-V307L and V307L tissue to unidirectional block in response to a premature stimulus applied during the refractory tail of a previous excitation wave. During such conduction block tissue is rendered more susceptible to re-entrant arrhythmia. Supplementary Figure S3 shows the percentage increase compared to the WT condition in the temporal vulnerable window to conduction block, measured at a location 5.0 mm away from the epicardial end of the 1D strand. The width of the vulnerable window was increased by 37% and 82% with respect to the WT in WT-V307L and V307L conditions, respectively.

### Investigating the arrhythmogenic substrate in SQT2 – 2D and 3D simulations with realistic geometry

**Figure 5:**
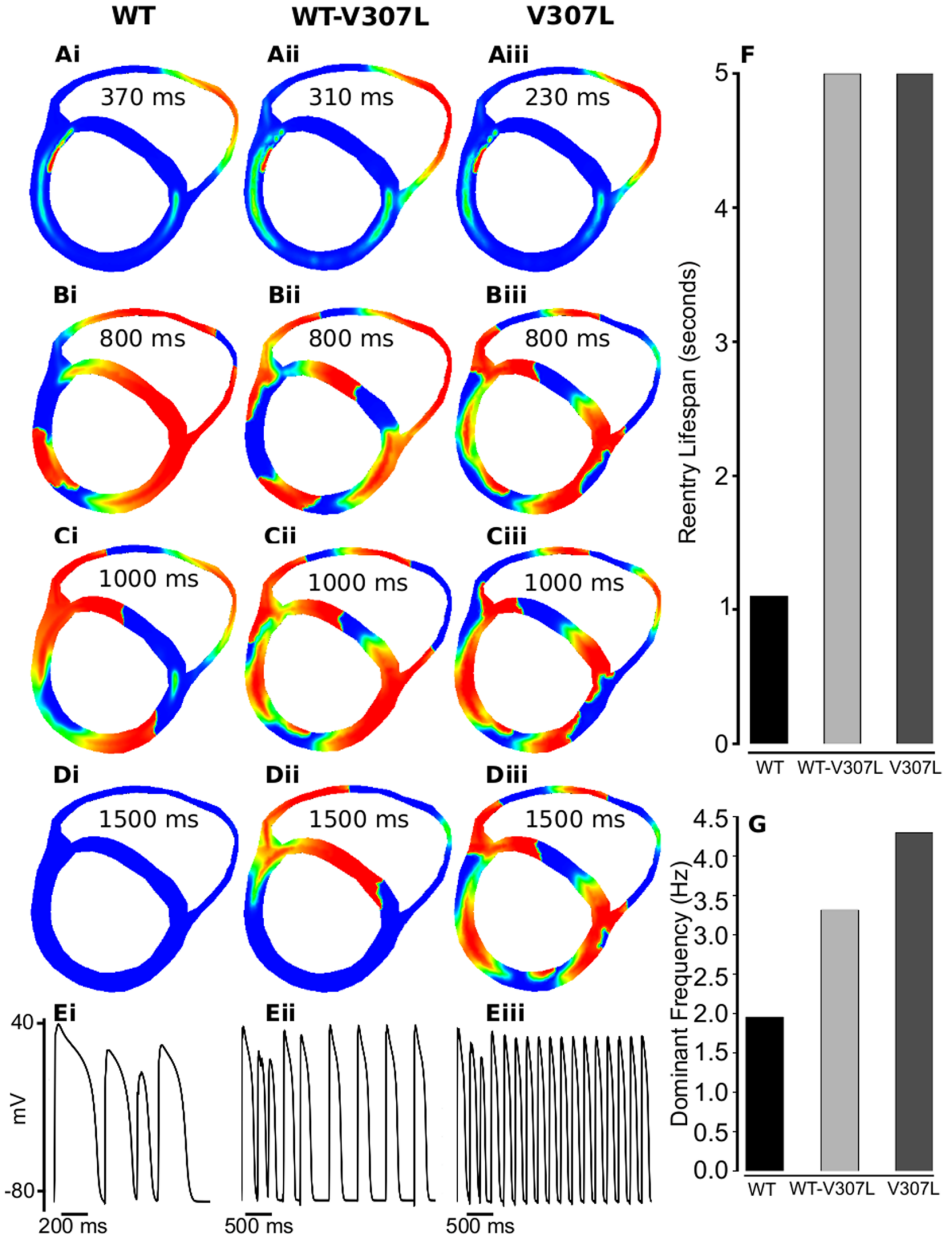
Snapshots of re-entry in 2D cross-section of ventricles. (**A**) Application of a premature S2 stimulus into the refractory and partially recovered tissue following excitation after a delay of 370 ms for WT (i), 310 ms for WT-V307L (ii), and 230 ms for V307L (iii) conditions. Snapshots of developed spiral waves at time, *t* = 800 ms (**B**), *t* = 1000 ms (**C**), and *t* = 1500 ms (**D**) in WT (i), WT-V307L (ii), and V307L (iii) conditions. (**E**) Time series of cellular APs recorded in the left ventricle in WT (i), WT-V307L (ii), and V307L (iii) conditions. Measured lifespan (**F**) and dominant frequency (**G**) of re-entrant spiral waves. Computed dominant frequencies are 1.96 Hz, 3.32 Hz, and 4.30 Hz for WT, WT-V307L, and V307L conditions, respectively.

In a realistic human ventricle cross-sectional slice (Fig. 5), we investigated the response of WT, WT-V307L and V307L tissue to a local premature stimulus applied within the left ventricular wall during the tissue’s vulnerable window (WT: 370 ms after the arrival of conditional wavefront; WT-V307L: 310 ms after the arrival of conditional wavefront; V307L: 230 ms after the arrival of conditional wavefront). The results of 2D simulations are shown in Fig. 5 (and Supplementary Videos S1–3). Following the premature stimulus, a re-entrant excitation wave was initiated within the left ventricular wall as shown in Fig. 5Ai–Di for WT, Fig. 5Aii–Dii for WT-V307L and Fig. 5Aiii–Diii for the V307L condition. The snapshots shown in Fig. 5A–D show subsequent conduction of the induced re-entrant excitation wave from the applied premature stimulus for the WT (Fig. 5Bi–Di), WT-V307L (Fig. 5Bii–Dii) and V307L (Fig. 5Aiii–Diii) conditions. Under the WT condition, the initiated re-entry self-terminated after 1.1 s (Fig. 5Di, Supplementary Video S1) but it persisted under the mutation conditions throughout the whole simulation period (5s) (WT-V307L: Fig. 5Bii–Dii, Supplementary Video S2; V307L: Fig. 5Biii–Diii, Supplementary Video S3).

The time course of an AP in the left ventricle is shown for the WT, WT-V307L and V307L conditions in Fig. 5Ei–Eiii respectively. Power spectrum analysis of the recorded whole-field averaged electrical activity from the tissue revealed a higher dominant frequency in the mutation conditions (3.32 Hz for WT-V307L and 4.30 Hz for V307L compared to the WT condition (1.96 Hz) (Fig. 5F). These 2D simulation results illustrate that the KCNQ1 V307L mutation increases tissue susceptibility to arrhythmogenesis by maintaining re-entrant excitation waves.

**Figure 6:**
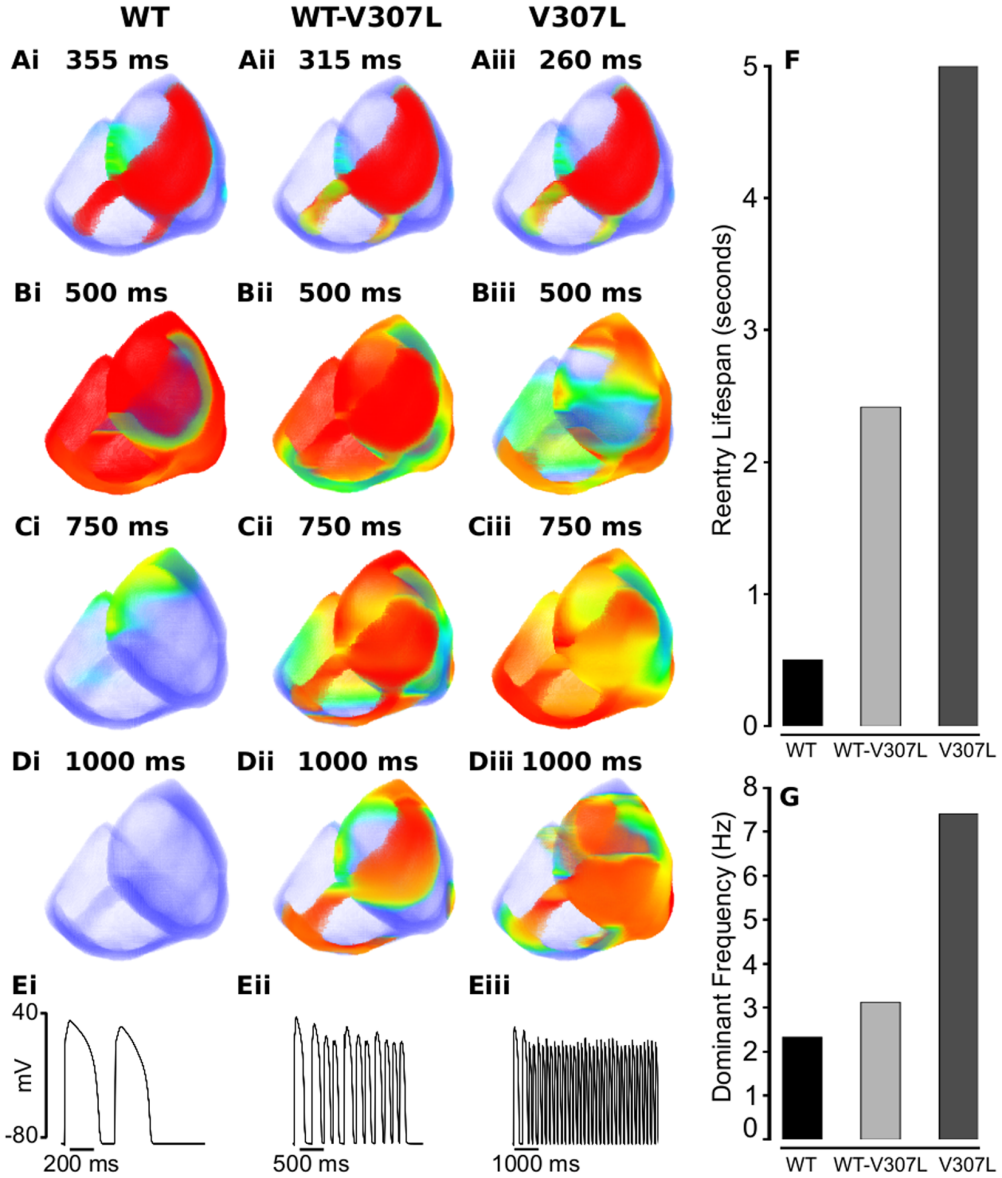
Snapshots of re-entry in 3D anatomical model of ventricles. (**A**) Application of a premature S2 stimulus in a local region during the refractory period of a previous excitation wave after a time delay of 355 ms for WT (i), 315 ms for WT-V307L (ii), and 260 ms for V307L (iii) conditions. Snapshots of developed scroll waves at time, *t* = 500 ms (**B**), *t* = 750 ms (**C**), and *t* = 1000 ms (**D**) in WT (i), WT-V307L (ii), and V307L (iii) conditions. (**E**) Time series of cellular APs recorded in the left ventricle in WT (i), WT-V307L (ii), and V307L (iii) conditions. Measured lifespan (**F**) and dominant frequency (**G**) of re-entrant spiral waves. Computed dominant frequencies of electrical activity recorded in the left ventricle are 2.34 Hz, 3.13 Hz, and 7.42 Hz for WT, WT-V307L, and V307L conditions, respectively.

Realistically, the ventricles are three-dimensional and have a much more complex anisotropic geometry compared to the 2D ventricular slice. Therefore, one cannot necessarily assume that sustained reentry in the 2D tissue model translates to similar activity in 3D tissue. Consequently, we performed further simulations with a 3D anatomical human ventricle geometry. The results are shown in Fig. 6, which shows snapshots of the evolution of re-entrant scroll waves (WT: Fig. 6Ai–Di, Supplementary Videos S4 and S5; WT-V307L: Fig. 6Aii–Dii, Supplementary Videos S6 and S7; V307L: Fig. 6Aiii–Diii, Supplementary Videos S8 and S9) developing as a response to a premature stimulus. For the WT condition, the scroll wave self-terminated with a lifespan of 0.5 s (Fig. 6F). However, under WT-V307L and V307L mutation conditions, the scroll wave broke up forming multiple re-entrant wavelets that self-terminated within 2.5 s in WT-V307L tissue but were sustained throughout the 5 s simulation period in V307L tissue (Fig. 6F). Power spectrum analysis of the registered pseudo-ECG shows the dominant frequency of ventricle excitation to be 2.34 Hz for the WT condition, 3.13 Hz for the WT-V307L mutation condition and 7.42 Hz for the V307L mutation condition (Fig. 6G). Figure 6Ei–Eiii show a recording of the evolution of the AP of a cell in the left ventricle for the WT, WT-V307L and V307L conditions.

### Investigating I_Ks_ as a potential therapeutic target in SQT2

**Figure 7:**
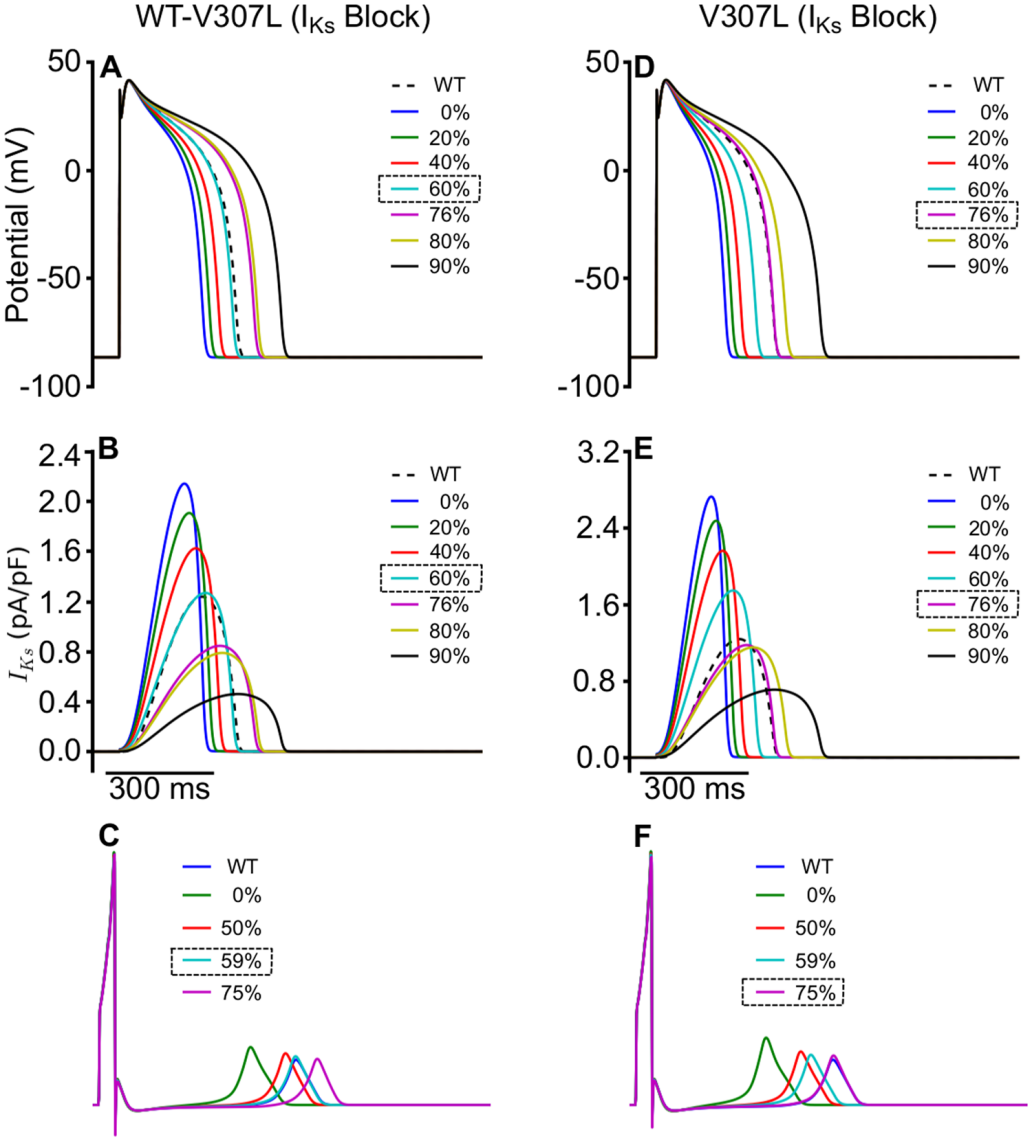
I_Ks_ blockade in single cell and 1D simulations. Action potentials in WT-V307L (**A**) and V307L (**D**) conditions under varying degrees of I_Ks_ blockade. The dashed line represents the WT and the boxed percentage represents the degree of I_Ks_ block required to normalize the APD under the respective mutation condition. I_Ks_ profiles corresponding to the APs shown in (**A**) and (**D**) are shown for WT-V307L (**B**) and V307L (**E**) conditions, respectively. Pseudo-ECGs corresponding to varying degrees of I_Ks_ blockade are shown in WT-V307L (**C**) and V307L (**F**) conditions. The blue line represents the WT and the boxed percentage represents the degree of I_Ks_ block required to normalize the QT interval under the respective mutation condition.

As a pseudo-pharmacological approach to treating patients with SQT2, we mimicked I_Ks_ channel blockade by drugs in order to determine the extent of blockade required to normalise the QT interval. To investigate this, maximal channel conductance of mutant I_Ks_ was reduced in the EPI, MIDDLE and ENDO single cell types. Figure 7A–E show the results for an EPI cell in the TNNP model under the WT-V307L (Fig. 7A–B) and V307L (Fig. 7D–E) conditions. In all three cell types, approximately 60% simulated I_Ks_ blockade was required to make the APD comparable to that of WT under the WT-V307L mutation condition while approximately 76% I_Ks_ blockade was necessary under the V307L mutation condition.

Using the intact 1D strand, we performed a similar investigation on the normalization of the QT interval. In tissue, due to the electrical coupling between cells via gap junctions, APD differences between different cell types are reduced. Thus, for the WT-V307L mutation condition, 59% I_Ks_ reduction (Fig. 7C) was needed to normalise the QT interval to that of WT while under the V307L mutation condition, approximately 75% I_Ks_ reduction was necessary (Fig. 7F). These results are similar to the single cell situation.

**Figure 8:**
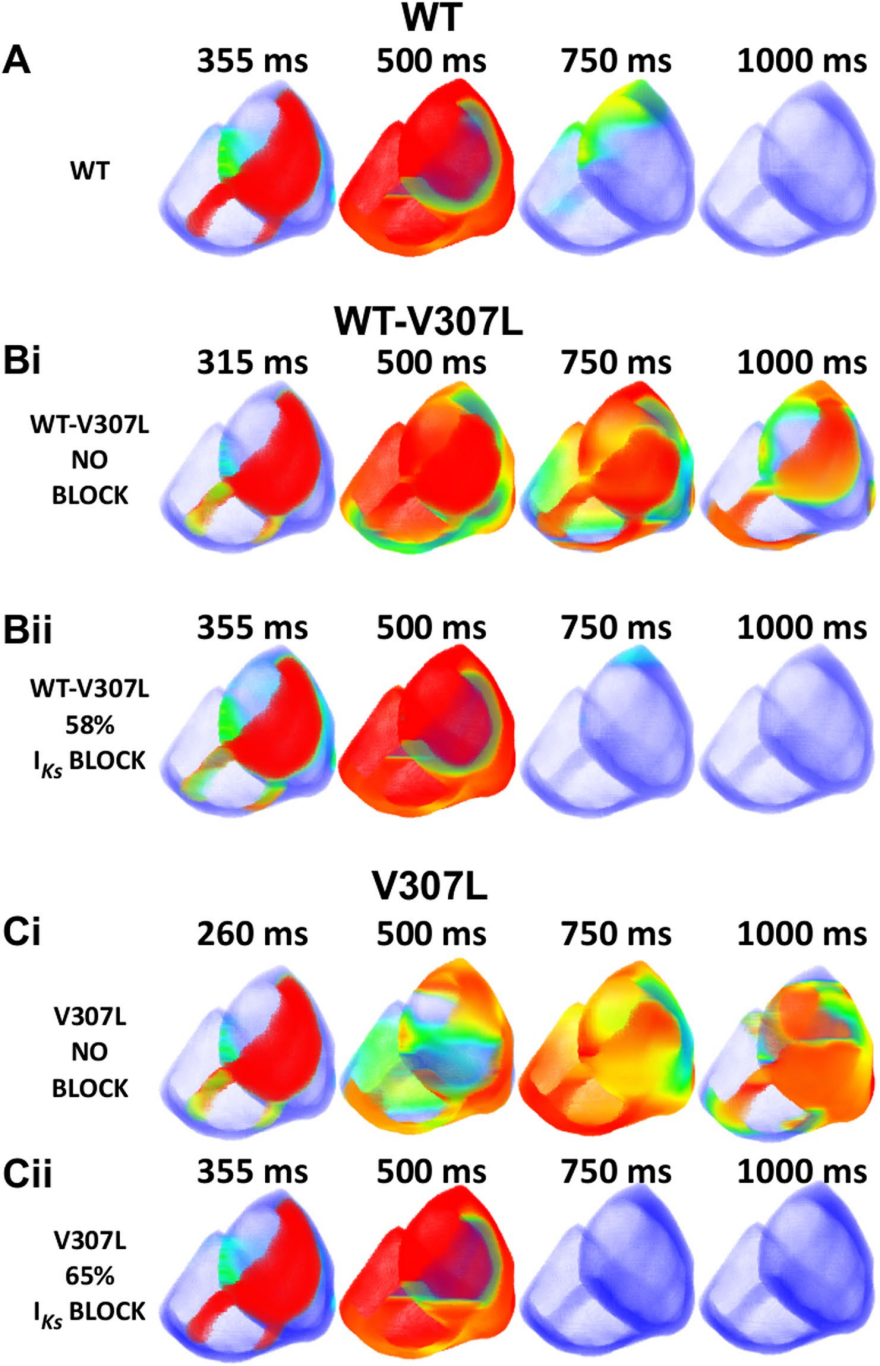
Termination of re-entry in 3D ventricle model by I_Ks_ blockade. (**A**) Application of a premature S2 stimulus in a local region of WT tissue during the refractory period (355 ms) leads to the development of a scroll wave (500 ms) that terminates in under 1000 ms. (**Bi**) Application of a premature S2 stimulus in a local region of WT-V307L tissue during the refractory period (315 ms) leads to the development of a scroll wave (500 ms) which persists beyond 1000 ms. (**Bii**) Application of a premature S2 stimulus in a local region of WT-V307L tissue with 58% I_Ks_ blockade during the refractory period (355 ms) leads to the development of a scroll wave (500 ms) that terminates in under 1000 ms. (**Ci**) Application of a premature S2 stimulus in a local region of V307L tissue during the refractory period (260 ms) leads to the development of a scroll wave (500 ms) which persists beyond 1000 ms. (**Cii**) Application of a premature S2 stimulus in a local region of V307L tissue with 65% I_Ks_ blockade during the refractory period (355 ms) leads to the development of a scroll wave (500 ms) that terminates in under 750 ms.

Finally, we investigated whether simulated I_Ks_ blockade could terminate re-entrant activity under the mutation conditions (Fig. 8) in the 3D anatomical human ventricles. Figure 8A shows the WT condition with a premature stimulus applied during the tissue’s vulnerable window at 355 ms. If I_Ks_ reduction under the mutation conditions normalises the QT interval, then the application of a premature stimulus at this same time (355 ms) should produce somewhat similar activity to WT. Figure 8Bi and Ci show WT-V307L and V307L mutant tissue with no I_Ks_ reduction, i.e. the pure heterozygote and homozygote mutant conditions respectively with a premature stimulus applied at 315 ms for WT-V307L and 260 ms for V307L leading to re-entrant activity that persists beyond the WT reentry lifespan. It transpired that 58% I_Ks_ reduction was sufficient to make the WT-V307L reentry lifespan (Fig. 8Bii) comparable to that of WT while 65% I_Ks_ reduction was adequate under the V307L mutation condition (Fig. 8Cii). These simulations illustrate the possibility of I_Ks_ as a relevant drug target to treat tachyarrhythmia in the SQT2 setting.

## Discussion

In this study, we have developed a MC model of I_Ks_ incorporating the SQT2 V307L KCNQ1 mutation in order to elucidate possible pro-arrhythmic effects of this *KCNQ1* gene mutation, and investigate *in silico* the possibility of I_Ks_ inhibition as a treatment for SQT2. The major findings of the present study are summarised as follows: (i) a novel biophysically-detailed MC model formulation that reproduces accurately the dynamic properties of the KCNQ1 V307L mutation; (ii) the V307L mutation abbreviates the APD and steepens the APD restitution curve; (iii) the V307L mutation shortens the QT interval, increases T wave amplitude and T_peak_ − T_end_ duration, all of which are concordant with clinical observations regarding the SQTS; (iv) significantly reduced ERP in all transmural cell types in the *KCNQ1* V307L mutation accelerates re-entrant excitation waves in human ventricles; (v) augmented membrane potential (δV) differences and transmural APD dispersion associated with the *KCNQ1* V307L mutation contributes to increased T-wave amplitude and increased temporal vulnerability to uni-directional conduction block by a premature excitation at some localised regions of the ventricles; (vi) I_Ks_ blockade is a potential therapeutic means of normalizing the QT interval and terminating re-entrant activity in the SQT2 setting. These results provide increased insight into understanding the causal link between the *KCNQ1* V307L mutation and QT interval shortening and tachyarrhythmia.

In the first report of the *KCNQ1* V307L mutation in a patient with the SQTS, Bellocq *et al*.^10^ used a Priebe-Beuckelmann ventricular cell AP model^22^ to demonstrate AP shortening. In our previous study^16^, we used the 2004 TNNP human ventricular AP cell model^23^ with modified HH I_Ks_ formulations reproducing V307L KCNQ1+KCNE1 kinetics available at that time to also demonstrate AP abbreviation, QT interval shortening, T wave morphology changes, reduced minimal substrate size for re-entry and reentrant activity in idealised 2D geometry. However, the current study is the first to reproduce the subsequently identified slower deactivation of V307L KCNQ1+KCNE1 I_Ks_ and to examine the mutation’s functional consequences in realistic 2D and 3D anatomical geometries. The present study is also the first to investigate the blockade of I_Ks_ as a potential pharmacological intervention for treating SQT2 patients.

The MC structure used in this study^24^ was previously employed in the study of O’Hara *et al*.^25^, in which effects of the “silent” Q357R KCNQ1 mutation in the long QT syndrome (LQT1) were probed *in silico*. That study^25^ found that multiple insults to the repolarization reserve were required to reproduce the complex arrhythmic phenotype associated with the Q357R mutation, highlighting the need for a more kinetically-accurate description of I_Ks_ channel kinetics than can be achieved with traditional HH formulations. This study builds on the approach employed by O’Hara *et al*.^25^, incorporating a detailed MC description of I_Ks_ kinetics associated with a KCNQ1 channelopathy into anatomically-detailed human ventricle tissue simulations.

The SQTS is associated with malignant tachycardias^2, 4, 10, 26^ and some patients present with ventricular fibrillation episodes^10, 27^ including the SQT2 proband^10^, who was successfully resuscitated from ventricular fibrillation. In our simulations we found that the *KCNQ1* V307L mutation reduces ERP at all rates (Fig. 3). This helps to maintain re-entrant activity in tissue, as it decreases the wavelength of ventricular excitation waves, allowing higher activation frequencies of re-entrant excitation waves. This is evident in the increased lifespan and dominant frequency associated with the WT-V307L and V307L mutation conditions in both 2D and 3D simulations (Figs 5 and 6). Furthermore, we found that the SQT2 mutation increases vulnerability to *initiation* of re-entrant activity through localized increases in transmural heterogeneity of membrane potential, which leads to an increased temporal window of vulnerability to uni-directional conduction (Supplementary Figure S3). These findings are qualitatively in accord with our previous study^16^ using the HH model of SQT2 with idealized tissue geometrical structures, demonstrating the elucidated arrhythmogenenic mechanisms of SQT2 are model independent and further solidify the causative link between *KCNQ1* V307L mutation and ventricular arrhythmogenesis.

The use of Implantable Cardioverter Defibrillators (ICDs) is the current preferred treatment for the SQTS^2, 4, 28, 29^. However, as the SQTS is characterised by tall and peaked T-waves, there is the risk of inappropriate shocks to the patient due to T-wave over-sensing^2, 4, 28^. Additionally, ICDs do not restore the QT interval to its normal duration and are not suitable for all patients (e.g. infants). Therefore, pharmacological alternatives that can restore the normal duration of the QT interval and offer protection from arrhythmias are being actively pursued^2, 4, 30, 31^. Hydroquinidine has been found to be effective in prolonging the QT interval and preventing episodes of VF, but its effect is greater in patients with identified SQT1 (hERG) mutations than in those without SQT1 mutations^32^. At present, there is no selective I_Ks_ blocker in clinical use. *In vitro* experiments have shown that although I_Ks_ is selectively blocked by chromanol 293B^33, 34^, the blocking potency of this agent is reduced for recombinant channels containing the V307L KCNQ1 mutation^17, 34^. In contrast, I_Ks_ channels incorporating the V307L KCNQ1 mutation were inhibited by the quinolone agent mefloquine with a similar potency to that for WT channels^17^.

Our simulations mimicked selective pharmacological block of the I_Ks_ channel in the SQT2 setting through reductions in I_Ks_ magnitude. These showed that in the heterozygotic WT-V307L KCNQ1 condition, a blockade of I_Ks_ by ~58% is sufficient to restore the QT interval to its original duration and make the tissue behave like WT tissue whereas under the V307L homozygote condition, a blockade of I_Ks_ by ~65% is sufficient to achieve the same result. In our simulations, a high degree of I_Ks_ block was required in the setting of SQT2 to ‘correct’ the cellular level APD and QT interval in tissue as well as to reduce tissue vulnerability and lifespan of reentry to those of normal tissue. This is consistent with the extent of I_Ks_ augmentation arising from SQT2 conditions, however. This is demonstrated in Supplementary Figure S13. In these simulations, increasing maximal I_Ks_ channel conductance in the WT condition by an amount equal to the inverse of that required to normalise the AP in SQT2 mutant conditions led to comparable shortening of the APD, and thus corresponded approximately to the amount of increased I_Ks_ due to SQT2 mutant conditions. Although simulated I_Ks_ block is a theoretical consideration (in the absence of clinically used selective I_Ks_ inhibitors), and involves a simple current reduction rather than modification of kinetics, our study is nonetheless the first to provide proof-of-concept that I_Ks_ block has the potential to be effective in the SQT2 setting. Furthermore, whilst the important role of I_Ks_ in repolarization reserve means that I_Ks_ blockade can lead to torsade de pointes^33, 35, 36^, this risk is likely to be greatest against a background of a normal, rather than abbreviated QT interval.

Inhibition of I_Ks_ as a potential therapeutic strategy was further tested in another form of SQT2 caused by the *KCNQ1* V141M mutation, which differs from the V307L mutation in that it induces a constitutively active voltage-independent current component^11^. Briefly, the MC was further modified to recapitulate kinetics of the V141M KCNQ1 mutation (shown in Supplementary Figure S11), including presence of a voltage-independent component, shifted voltage dependence of activation to less depolarized potentials, and significantly slowed deactivation^37^. We found that the heterozygous WT-V141M condition produced a significant shortening of the APD and QT interval, consistent with the SQTS phenotype. Simulation of a substantial degree of I_Ks_ block (79%) resulted in normalization of the AP, as shown in Supplementary Figure S12. These supplementary simulations help to justify and validate our modelling approach, whilst providing further evidence that I_Ks_ block may, in principle, be an effective therapeutic strategy in the setting of SQT2.

In order to determine whether or not the principal results obtained in this study pertaining to the *KCNQ1* V307L mutation were model-dependent, single cell and 1D simulations performed in the TNNP model were also carried out in the more recently-developed O’Hara-Rudy dynamic (ORd) human ventricular cell model^38^. At the single cell level, the major findings reported using the TNNP model were matched qualitatively by the ORd model, i.e. the SQT2 mutations significantly reduced the APD, affecting transmural regions of the ventricles differentially (Supplementary Figure S4), and increased maximum slope of APD restitution (Supplementary Figure S5). In the ORd model intact 1D strand, the MC formulation of the *KCNQ1* V307L mutation accurately reproduced QT interval shortening and an increase in T-wave amplitude – two hallmarks of the SQTS (Supplementary Figure S6)^2, 4–7^. The underlying mechanisms contributing to increased T-wave amplitude due to the SQT2 mutations were the same as in the TNNP model, i.e. augmented maximal membrane potential difference, δV, between coupled cells in tissue (Supplementary Figure S7) due to increased spatial gradient of APD across the transmural strand (Supplementary Figure S8). Furthermore, tissue temporal vulnerability to uni-directional conduction in the ORd model 1D transmural strand (measured at the MIDDLE-EPI border) was increased by the SQT2 mutations (Supplementary Figure S9).

I_Ks_ reductions of 50% and 65% in WT-V307L and V307L conditions respectively were required to normalize the APD in single cell ORd model simulations (Supplementary Figure S10). These values are comparable to the TNNP values of 60% and 76%. Similar to the single cell situation, the degrees of I_Ks_ blockade necessary to normalise the QT interval in SQT2 mutation conditions in the ORd model were slightly lower but still fairly close to those in the TNNP model. The model independence of these results is significant. Obtaining qualitative agreement between two distinct human ventricle cell models shows that the fundamental mechanisms by which the SQT2 mutations affect ventricular electrophysiology are the same even when there are differences in ion channel kinetics, as in the TNNP and ORd models.

Limitations of the main human ventricular cell model used (TNNP 2006) have been discussed in detail elsewhere^18, 20, 39^. In the multicellular tissue model - due to a lack of detailed experimental data - the proportion of each region comprised each distinct cell type and intercellular electrical coupling was chosen to produce a positive T-wave and a conduction velocity of a planar solitary excitation wave close to experimental data, similar to those used in other studies^16, 21, 40^. The heterozygote formulation used in this study relies on the simplifying assumption that V307L I_Ks_ behaves similar to a 50:50 mixture of WT and mutant channels. The channel population may be more complex in reality, with each channel comprising both WT and mutant KCNQ1 subunits. That said, our previous study^16^ investigated the effects of varying mutant subunit composition in SQT2 based on different expression/co-expression ratios used in experiments in the original paper describing the V307L KCNQ1 mutation^10^, and found that the degree of APD and QT interval shortening increased progressively with the level of V307L expression. Thus, the simplifying assumption adopted in the present study is unlikely to affect adversely the simulation results: our heterozygote formulation reproduced QT interval shortening and increased T wave amplitude associated with the SQTS phenotype^2, 4–7^.

Care must be exercised in the interpretation of results from the 2D model, as it is based on a single slice of the ventricle wall. Although we considered anisotropic intracellular electrical coupling in the 2D and realistic anatomical structure, the 2D model nevertheless represents only a cross-sectional slice through the ventricle; it therefore lacks features of an anatomically realistic 3D ventricular geometry such as the irregular thickness of the wall structure across the entire geometry, layered structure of ventricular tissue and more realistic anisotropy, all of which could influence maintenance of ventricular arrhythmias in the V307L mutation conditions. These potential limitations predicated our additional use of 3D simulations. Though the 3D model incorporates ‘realistic’ ventricular anatomical geometry, it lacks inclusion of a Purkinje fibre network, which may play a role in arrhythmogenesis in the SQTS^40^. In addition, the tissue models in this study do not consider the effect of cardiac mechanics on tissue geometry, which feasibly might influence re-entry^41, 42^, particularly for SQTS patients^43–45^.

Whilst it is important that both assumptions and potential limitations of the models used in this study are made explicit, these do not influence fundamentally the conclusions that can be drawn on likely mechanisms by which the *KCNQ1* V307L mutation facilitates arrhythmia induction and maintenance, and the possibility of I_Ks_ inhibition as pharmacological modulation in the setting of SQT2. Moreover, it is striking that in our simulations, despite differing levels of complexity, the 2D and 3D simulations yielded qualitatively if not quantitatively similar findings in terms of identifying mechanisms that can account for increased arrhythmia susceptibility in SQT2. This highlights the likely importance of the pro-arrhythmic mechanisms identified in this study.

The simulations in this study substantiate that the *KCNQ1* V307L mutation is causally linked to QT interval shortening and increased tissue vulnerability to arrhythmogenesis in this form of the SQTS. In addition, we have shown that I_Ks_ blockade normalises the QT interval and successfully terminates re-entry during tachyarrhythmias. Thus, the findings of this study provide a comprehensive explanation for clinical consequences of this form of the SQTS in terms of abbreviation of repolarisation and susceptibility to arrhythmia and it provides a potential pharmacological approach in designing therapeutic interventions for this form of the SQTS. It is notable that the more recently reported R259H^13^ and F279I^46^ KCNQ1 SQT2 mutations produce similar changes to recombinant channel I_Ks_ to those of V307L KCNQ1. The results reported here may therefore also have relevance to SQT2 associated with these mutations. Additionally, the multi-scale ventricular models developed and employed in this study may have further utility for probing the basis of arrhythmia, both in other forms of the SQTS and other repolarisation disorders.

## Methods

### Development of I_Ks_ Markov chain model

The MC model of I_Ks_ incorporating the V307L KCNQ1 mutation was based on the work of Silva and Rudy^24^, who developed it using experimental data at physiological temperature (37 °C) including data from human ventricular myocytes representing I_Ks_ activation and deactivation kinetics (MC scheme shown in Supplementary Figure S1). The model was modified to reflect the experimentally observed kinetic properties of WT and V307L-mutant “I_Ks_” (KCNQ1+KCNE1) channels. These kinetic properties include: (i) the profound leftward shift (−36 mV) of channel activation for the V307L mutation compared to WT^17^, (ii) slower channel deactivation in the V307L mutation^17^, (iii) the increased repolarising current during ventricular AP clamp^17^, and (iv) accelerated activation as seen with the V307L mutation^10, 17^.

To obtain the transition rates of the MC model that reproduced the experimentally observed kinetic properties of WT and V307L, we simulated the experimental current-voltage (I-V) relationships for WT and V307L KCNQ1 + KCNE1 using the voltage clamp protocol from El-Harchi *et al*.^17^ (Fig. 1Aii–Bii). The membrane potential was held at −80 mV and then depolarised briefly to −40 mV for 50 ms, followed by 3 s depolarisations to a range of potentials from −70 mV to +60 mV (in 10 mV increments); finally, tail currents were elicited by repolarisation to −40 mV for 5 s. The currents obtained at the end of the depolarising steps were normalised and compared to experimental data (Fig. 1Aiii–Biii). The elicited current traces from the voltage clamp protocol are shown in Fig. 1Ai–Bi.

By minimising the least-squared difference between the experimental data and the simulation, the variables representing the transition rates that produced the best fit and behaviour of macroscopic currents relative to the experimental data were obtained (Fig. 1Aiii–Biii). The minimisation was performed using the Nelder-Mead Simplex algorithm^47^. Relative current proportions for WT and V307L *KCNQ1* conditions were then scaled using relative proportions of peak I_Ks_ obtained from AP clamp experiments^17^.

### AP clamp and model validation

The MC model was validated by comparing simulated results with those observed experimentally during ventricular AP clamp (Fig. 1Aiv–Biv). The same digitised ventricular AP used to generate the experimental AP clamp^17^ was used in the simulation.

### Heterozygote formulation

To mimic the heterozygous state of the proband, we constructed a heterozygous formulation (WT-V307L) consisting of 50% WT and 50% V307L channels. We used this formulation to also investigate the effects of the KCNQ1 V307L mutation in this heterozygous condition.

### Cellular and tissue models

The developed I_Ks_ MC model was incorporated into the 2006 version of the TNNP^20^ model of the human ventricular cell AP for consistency with our previous *in silico* studies on the SQTS^18, 19, 44^. Single cell models were then incorporated into 1D, 2D and 3D multicellular models with a monodomain tissue representation^18, 19, 48^ of realistic human ventricular geometries. The realistic human geometries were reconstructed as described previously^18, 19^ with a spatial resolution of 0.2 mm. The anatomical model was segmented into three distinct regions: 25% EPI, 35% MIDDLE and 40% ENDO cells. These proportions are similar to those used in other studies^16, 18, 21, 40^ and were chosen as they produced a positive T-wave on the ECG under the WT condition.

Further details regarding multi-scale model development, including methods for simulating the pseudo-ECG, protocols used for measuring restitution of the APD and ERP, initiation of re-entrant excitation waves, dynamics of re-entry, and numerical methods have been documented in detail in our previous studies^16, 18, 19, 49^ and are provided in the Supplement.

## Electronic supplementary material

Supplementary Information

Video S1

Video S2

Video S3

Video S4

Video S5

Video S6

Video S7

Video S8

Video S9
