# Supplementary material for: In silico investigation of a KCNQ1 mutation associated with short QT syndrome
Source: Sci Rep. 2017 Aug 16;7:8469. doi: 10.1038/s41598-017-08367-2 (PMC5559555; doi:10.1038/s41598-017-08367-2)
Supplement: Supplementary file 1 — Supplementary Information [file 41598_2017_8367_MOESM1_ESM.pdf]

Data supplement

In silico investigation of a *KCNQ1* mutation associated with short QT syndrome

Ismail Adeniran[1,+], Dominic G. Whittaker[1,+], Aziza El Harchi[2], Jules C. Hancox[1,2,*] and Henggui Zhang[1,3,4*]

[1]Biological Physics Group, School of Physics & Astronomy, The University of Manchester, Manchester, M13 9PL, UK

[2]School of Physiology, Pharmacology and Neuroscience, Biomedical Sciences Building, University Walk, Bristol BS8 1TD, UK

[3] School of Computer Sciences and Technology, Harbin Institute of Technology, Harbin, China

[4] Space Institute of Southern China, Shenzhen, China

*Correspondence to henggui.zhang@manchester.ac.uk or jules.hancox@bristol.ac.uk

[+]these authors contributed equally to this work

Supplementary methods

Formulation of I_{Ks}

Figure S1 shows the state transition diagram of the I_{Ks} Markov chain (MC) model. To understand the state transition diagram, it is useful to mention the work of Zagotta *et al.* [1,2] on Shaker K^+ channels. They proposed a model with four subunits with identical activation rates. Each subunit undergoes two conformational transitions; say T1 (rest state) and T2 (intermediate state) before reaching the activated state. In 2003, Silverman *et al.* [3] provided experimental validation of this two-stage voltage sensor transition.

The I_{Ks} MC model shown in Figure S1 is based on this two-stage voltage sensor transition principle. It consists of two open states (O1 and O2) and 15 closed states (C1 to C15). There are three zones: zone 2 (green) consists of those closed states with voltage sensors that have not completed the first transition; zone 1 (blue) consists of those closed states with voltage sensors that have completed the first transition; and the open zone (red) which represents the activated state. Left to right transitions represent a voltage sensor movement from its rest state (T1) to its intermediate state (T2) while top to bottom transitions represent a voltage sensor movement from its intermediate state (T2) to its activated state. For example, C3 has two voltage sensors in T1 and two in T2; C7 has two voltage sensors in T1, one in T2 and one in the activated state. For more details, see [4,5].

Transition rates of the MC model in wild-type (WT) and SQT2 mutation conditions were obtained using the Nelder-Mead simplex algorithm [6], as described in the main manuscript. Model parameters are shown in Figure S1.

Single cell models and AP simulations

The MC formulations of I_{Ks} were incorporated into the 2006 version of the TNNP human ventricular cell model [7], as updated by Xia *et al.* [8]. This model is particularly well-suited to the study of re-entrant arrhythmias and has employed in our previous studies [9,10]. In single cell models the cell membrane is modelled as a capacitor connected in parallel with variable resistors and batteries which represent different ionic currents, exchangers, and pumps. The cellular membrane potential is described with the following ordinary differential equation:

$$\frac{dV}{dt} = -\frac{I_{ion} + I_{stim}}{C_m} \qquad (1)$$

where V is the membrane potential, t is time, I_{ion} is the total ionic current, I_{stim} is an externally-applied stimulus current, and C_m is the cell capacitance per unit surface area.

Supplementary Equation 1 was integrated with a time step of 0.02 ms using the forward Euler method. The Hodgkin-Huxley equations for gating variables of various time-dependent ionic currents were integrated using the Rush-Larsen scheme [3], while the I_{Ks} MC model developed in this study was integrated with the forward Euler method.

Measurement of APD and ERP

We defined action potential duration (APD) as the action potential duration at 90% repolarization (APD_{90}). APs were elicited with a S1-S2 protocol comprising 10 S1 stimuli at a frequency of 1 Hz and a single S2 stimulus. Following the AP evoked by the final S1 stimulus, the S2 stimulus was applied at varying diastolic intervals (DI). The APD restitution (APD-R) curve was computed by varying the DI and plotting the APD_{90} evoked by the S2 stimulus against DI. At varying basic cycle lengths (BCL), the effective refractory period (ERP) was measured as the minimum DI for which the overshoot of the AP evoked by the S2 stimulus attained 80% amplitude of the AP evoked by the 10th S1 stimulus at each BCL. The ERP restitution (ERP-R) curve was generated by plotting the measured ERP against BCL.

Heterogeneous multicellular ventricular tissue models

Initiation and propagation of APs in multicellular tissue models was modelled using a

monodomain approach [11]:

$$C_m \frac{dV}{dt} = -\left(I_{ion} + I_{stim}\right) + \nabla \cdot \left(D \nabla V\right) \quad (2)$$

where D is the global conductivity tensor (diffusion coefficient) describing the tissue conductivity, and all other parameters retain their previous definitions. D is defined as:

$$D = \Lambda \Omega \Lambda^T$$

where Λ is a matrix of perpendicular unit vectors as columns (α_f in fibre direction, α_s in sheet direction and α_c in cross-sheet direction). Ω is the conductivity tensor expressed in the basis formed by the three perpendicular unit vectors:

$$\Omega = \begin{bmatrix} \sigma_f & 0 & 0 \\ 0 & \sigma_s & 0 \\ 0 & 0 & \sigma_c \end{bmatrix}$$

where σ_f is the fibre direction conductivity, σ_s is the sheet direction conductivity and σ_c is the cross-sheet direction conductivity. An entry in D is thus given by:

$$D_{ij} = \alpha_f^i \alpha_f^j \sigma_f + \alpha_s^i \alpha_s^j \sigma_s + \alpha_c^i \alpha_c^j \sigma_c$$

for $i, j = 0,1,2$.

For one-dimensional (1D) simulations a single fibre mesh of 100 nodes with 0.15 mm spacing was used, with each node representing a 150-μm cylindrical cell, giving a total length of 15 mm in good agreement with human transmural ventricle widths [12,13]. The transmural strand comprised 25 endocardial (ENDO) cells, 35 middle (MIDDLE) cells, and 40 epicardial (EPI) cells, with corresponding lengths of 3.75 mm, 5.25 mm, and 6 mm, respectively. This chosen proportion for each region is similar to that used in other studies [14–16], and produced a positive T-wave on the ECG under WT conditions.

There is ongoing debate on the existence of MIDDLE cells in the human ventricular myocardium. Some studies report no transmural difference in AP duration (APD) across the ventricular wall [17] while others have reported their presence [13,18–20]. MIDDLE cells have recently been found clustered in islands in the deep sub-endocardium of the human heart [19,20]. Hence, our

multicellular models incorporated MIDDLE cells, consistent with other reports from our laboratory [9,10].

The diffusion coefficient, *D*, which describes the spread of membrane potential in a reaction-diffusion system, was set to 0.001 cm^2/ms as in our previous studies [9,10], giving a conduction velocity (CV) of 65 cm/s through the strand, close to the 70 cm/s CV measured along the fibres in human myocardium [21]. *D* was homogenous except for a 5-fold decrease at the border of the EPI-MCELL regions [13,16].

To initiate an excitation wave in the 1D transmural strand, a supra-threshold stimulus was applied in the ENDO region. The CV was calculated from nodes one-quarter and three-quarters of the way across the strand as the quotient of the distance travelled and the difference in activation times (with activation time of each cell defined as the time of maximum upstroke velocity).

The realistic two-dimensional (2D) geometry used was employed in our previous studies [9,10], and is a transverse cross-sectional slice taken from the middle of the 3D ventricular geometry reconstructed by DT-MRI with spatial resolution 0.2 mm, segmented into distinctive ENDO, MIDDLE, and EPI regions. The geometry incorporated fibre anisotropy as in Seemann *et al.* [22], with intracellular conductivities set to 0.3 and 0.1 mS mm^{-1} in the fibre and cross-fibre directions, respectively.

Three-dimensional (3D) simulations were performed using an anatomical human ventricle geometry reconstructed using DT-MRI [23], taken from a healthy 30 year-old male. The geometry, which includes anisotropic fibre orientation, has a total of approximately 24.2 million cells. Both left and right ventricles are segmented into distinctive ENDO, MIDDLE, and EPI regions [23]. Activation sites were determined empirically across the walls of the ventricles, and validated through reproduction of the activation sequence and QRS complex in the measured 64-lead ECG [16]. Intracellular conductivities, taken from the work of Klepfer *et al.* [24], were set to 0.3, 0.1, and 0.3125 mS mm^{-1} in the fibre, sheet, and cross-sheet directions, respectively.

In multicellular simulations, equation 2 was solved using a Strang splitting scheme [25] and Crank-Nicholson scheme in time, together with Lagrangian Q1 finite elements in the spatial direction using the deal.II adaptive finite element library [26]. As the Strang-splitting scheme is accurate to the second-order and the Crank-Nicholson time-stepping scheme is unconditionally stable and second-order accurate with respect to time [27], the resulting solution is second-order accurate. Further details of numerical methods can be found in our previous studies [9,10].

Computing the pseudo-ECG

Pseudo-ECGs were calculated using the method outlined by Gima and Rudy [16], i.e.

$$\phi_e(x',y',z') = \frac{\alpha^2\sigma_i}{4\sigma_e}\int(-\nabla V_m)\cdot\left[\frac{1}{r}\right]dx \qquad (3)$$

$$r = \sqrt{(x-x')^2 + (y-y')^2 + (z-z')^2} \qquad (4)$$

where ϕ_e is a far-field unipolar potential, σ_e and σ_i are the extracellular and intracellular conductivities, respectively, α is the radius of the transmural fibre, and r is the distance from a source point (x,y,z) to a field point (x',y',z'). Using this method, the pseudo-ECG was recorded as ϕ_e at a position located 2.0 cm away from the epicardial end of the strand.

Measurement of temporal vulnerability window

Following propagation of a wavefront in cardiac tissue is a refractory tail. Applying a test stimulus in the 1D transmural strand after wave propagation can result in one of three outcomes: (i) bi-directional conduction block if the test stimulus is applied too early and tissue is still refractory in both directions; (ii) bi-directional conduction if the test stimulus is applied too late and tissue has recovered in both directions; (iii) uni-directional conduction block if tissue is still refractory *only* in one direction, allowing wave propagation in anterograde or retrograde direction but not both. The period of time during which uni-directional conduction can be evoked is the vulnerable window (VW) [9,10].

A train of 10 S1 stimuli were applied at the endocardial end of the 1D strand (1Hz, spatial size: 0.4 mm, amplitude: -52 pA/pF, duration: 1 ms) in order to evoke a propagating wave. Following a time delay (ΔT) after the 10th S1 stimulus, an S2 stimulus with the same duration and amplitude as the S1 stimulus was applied to a 0.4 mm region of the strand. The upper (T1) and lower (T2) bounds of ΔT during which excitation waves evoked by the S2 stimulus propagated uni-directionally in the strand were determined. The width (T1-T2), which provides a measure of the temporal vulnerability of the tissue, was computed across the strand.

Initiation of re-entry in 2D and 3D anatomical human ventricle geometries

In the 2D cross-sectional slice ventricle geometry, multiple S1 stimulus sites were chosen which recreated the activation sequence observed by Durrer *et al.* [28]. Re-entry was initiated by applying an S2 stimulus in the left ventricular endocardium during the vulnerability window to uni-directional conduction, leading to formation of re-entrant excitation waves.

3D scroll waves were also initiated using an S1-S2 protocol. Again, multiple S1 stimulus sites were chosen to produce an activation pattern across the ventricles in agreement with experimental measurements [28]. The S2 stimulus was applied during the refractory tail of the S1 stimulus, at a site

covering a small region of the left ventricular epicardium and a fraction of the right ventricular outflow tract, resulting in formation of re-entrant scroll waves.

Comparison with an alternative human ventricular cell model

Comparative simulations were carried out using the 2011 O'Hara-Rudy dynamic (ORD) human ventricle cell model [29]. Measurement of APD, QT interval, vulnerability window, etc. was carried out using the same protocols described in the TNNP model, in single cell and 1D strand ORd models.

The TNNP and ORd models are derived from different experimental data sets [7,29], and have been shown to have different strengths and weaknesses [30–32]. A previous modelling study, which studied the effects of the familial atrial fibrillation S140G KCNQ1 mutation on ventricular repolarization, reported a large discrepancy between responses of the TNNP and ORd models to that gain of function mutation to I_{Ks} channels [33]. Such huge model differences are somewhat diminished in this study, as a MC formulation of I_{Ks} with significantly different kinetics to the original ORd model I_{Ks} (HH formulation) was employed, and the conductance of I_{Kr} was also modified, to match experimental recordings from human ventricular myocytes [34]. This elevated the role of I_{Ks} in ventricular repolarisation and resulted in a more accurate shortening of the QT interval by the SQT2 mutant forms in the 1D ORd model strand.

Simulation of the *KCNQ1* V141M mutation in SQT2

Using the experimental data of Restier *et al.* [35], an I_{Ks} model was developed for the V141M mutation in KCNQ1, first described by Hong *et al.* [36]. As the experimental data was acquired at room temperature, a Q_{10} correction value of 3.5 [37,38] was applied in order to represent kinetics at body temperature. The I-V relation, steady state activation, and current trace recordings were used to constrain kinetic parameters during optimization, which was performed using a bounded Nelder-Mead simplex algorithm described by Moreno *et al.* [39]. An additional parameter, ξ, was introduced to account for the voltage-independent constitutively active component of I_{Ks} observed in *KCNQ1* V141M mutant channels [35,36]. A heterozygote formulation (WT-V141M) was constructed as for WT-V307L, using a 50:50 mix of WT and mutant I_{Ks} channels. 1D simulations were conducted using the same transmural strand model described for the V307L mutation.

Supplementary figures

I_{Ks} formulation

$$I_{Ks} = G_{Ks} \cdot O_{Ks} \cdot (V_m - E_{Ks})$$

$$G_{Ks} = 0.779 \cdot \left(1 + \frac{0.6}{1 + \left(\frac{3.8 \cdot 10^{-5}}{[Ca^{2+}]_i}\right)^{1.4}}\right)$$

$$O_{Ks} = O_1 + O_2$$

$$E_{Ks} = \frac{R \cdot T}{F} \cdot \log \frac{[K^+]_o + P_{Na/K} \cdot [Na^+]_i}{[K^+]_i + P_{Na/K} \cdot [Na^+]_i}$$

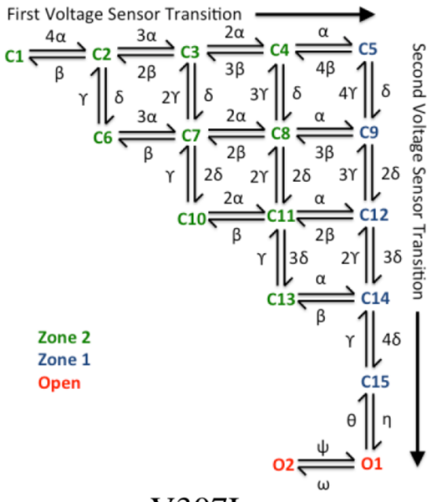

WT

$$\alpha = 5.56 \cdot 10^{-5} \cdot \exp\left\{3.61 \cdot 10^{-1} \cdot \frac{Vm \cdot F}{R \cdot T}\right\}$$

$$\beta = 8.25 \cdot 10^{-6} \cdot \exp\left\{-9.23 \cdot 10^{-2} \cdot \frac{Vm \cdot F}{R \cdot T}\right\}$$

$$\gamma = 3.78 \cdot 10^{-4} \cdot \exp\left\{8.68 \cdot 10^{-1} \cdot \frac{Vm \cdot F}{R \cdot T}\right\}$$

$$\delta = 1.32 \cdot 10^{-4} \cdot \exp\left\{-3.30 \cdot 10^{-1} \cdot \frac{Vm \cdot F}{R \cdot T}\right\}$$

$$\theta = 6.10 \cdot 10^{-4}$$

$$\eta = 1.95 \cdot 10^{-3} \cdot \exp\left\{-4.81 \cdot 10^{-1} \cdot \frac{Vm \cdot F}{R \cdot T}\right\}$$

$$\psi = 6.25 \cdot 10^{-4} \cdot \exp\left\{1.27 \cdot 10^{-0} \cdot \frac{Vm \cdot F}{R \cdot T}\right\}$$

$$\omega = 3.50 \cdot 10^{-4} \cdot \exp\left\{-6.79 \cdot 10^{-1} \cdot \frac{Vm \cdot F}{R \cdot T}\right\}$$

V307L

$$\alpha = 2.52 \cdot 10^{-5} \cdot \exp\left\{3.61 \cdot 10^{-1} \cdot \frac{Vm \cdot F}{R \cdot T}\right\}$$

$$\beta = 2.51 \cdot 10^{-5} \cdot \exp\left\{-9.23 \cdot 10^{-2} \cdot \frac{Vm \cdot F}{R \cdot T}\right\}$$

$$\gamma = 1.71 \cdot 10^{-3} \cdot \exp\left\{8.68 \cdot 10^{-1} \cdot \frac{Vm \cdot F}{R \cdot T}\right\}$$

$$\delta = 6.45 \cdot 10^{-4} \cdot \exp\left\{-3.30 \cdot 10^{-1} \cdot \frac{Vm \cdot F}{R \cdot T}\right\}$$

$$\theta = 3.40 \cdot 10^{-3}$$

$$\eta = 4.75 \cdot 10^{-4} \cdot \exp\left\{-4.81 \cdot 10^{-1} \cdot \frac{Vm \cdot F}{R \cdot T}\right\}$$

$$\psi = 1.93 \cdot 10^{-3} \cdot \exp\left\{1.27 \cdot 10^{0} \cdot \frac{Vm \cdot F}{R \cdot T}\right\}$$

$$\omega = 4.52 \cdot 10^{-4} \cdot \exp\left\{-6.79 \cdot 10^{-1} \cdot \frac{Vm \cdot F}{R \cdot T}\right\}$$

Figure S1: Markov I_{Ks} formulation. A summary of the I_{Ks} formulation used, with transition rate equations in WT and V307L conditions and corresponding Markov chain schematic diagram. Parameters are defined as follows: V_m is the membrane potential; G_{Ks} is the channel conductance; $[Ca^{2+}]_i$ is the intracellular calcium concentration; E_{Ks} is the reversal potential; R is the molar gas constant; T is the temperature; F is the Faraday constant; $[K^+]_o$ and $[K^+]_i$ are the extracellular and intracellular potassium concentrations, respectively; $[Na^+]_o$ and $[Na^+]_i$ are the extracellular and intracellular sodium concentrations, respectively; and $P_{Na/K}$ is the Na^+:K^+ permeability ratio.

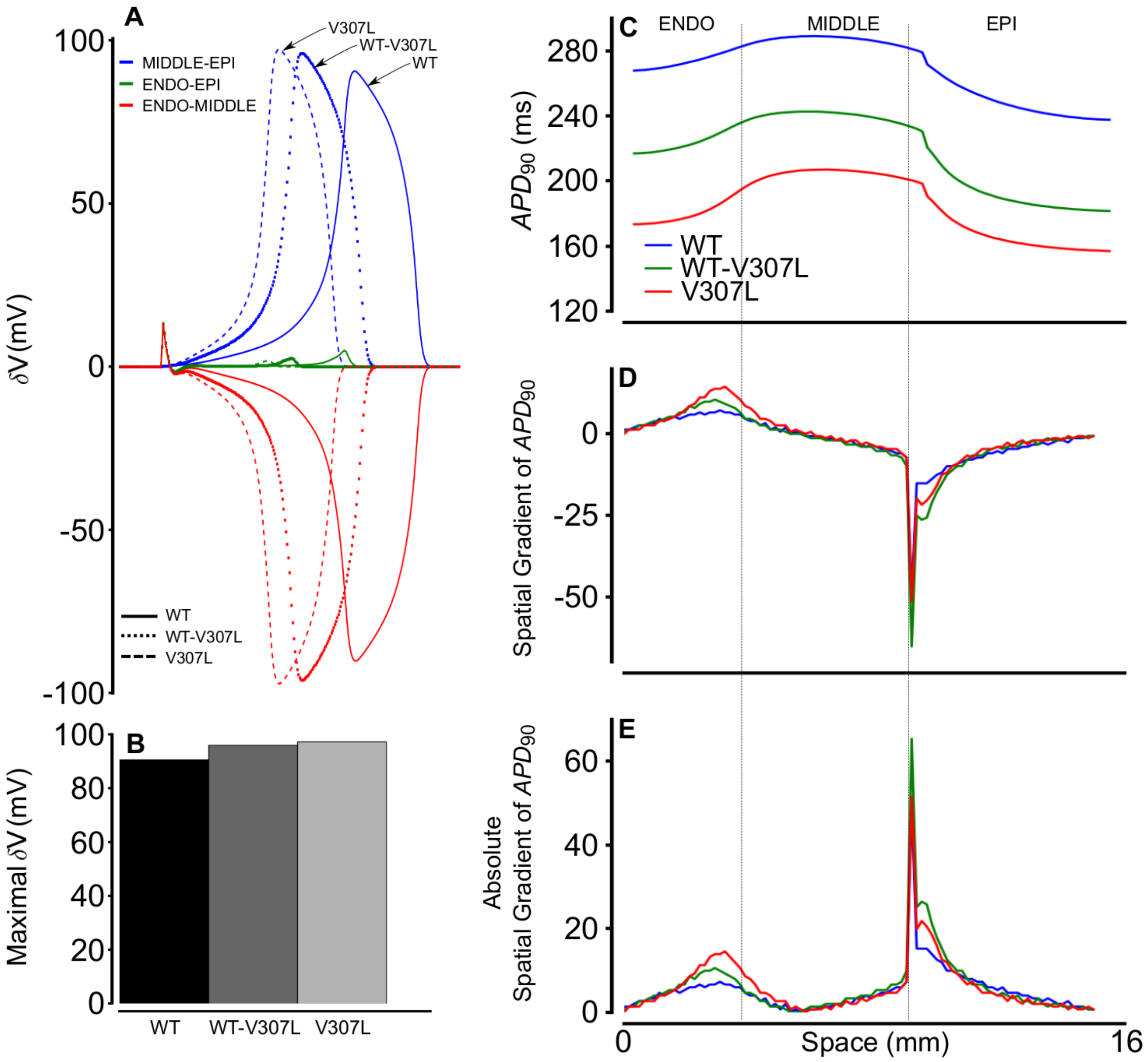

Figure S2: Membrane potential heterogeneity and spatial dispersion of APD_{90}. A plot of membrane potential heterogeneity (δV) in time (A) for WT (solid lines), WT-V30L (dotted lines), and V307L (dashed lines) conditions between the MIDDLE-EPI regions (blue), ENDO-EPI regions (green), and ENDO-MIDDLE regions (red). Maximal δV during repolarisation between MIDDLE-EPI cells (B). Transmural dispersion of APD_{90} across the 1D strand (C) in WT (blue), WT-V307L (green), and V307L (red) conditions. Measured (D) and absolute (E) spatial gradient of APD_{90} in the 1D transmural strand.

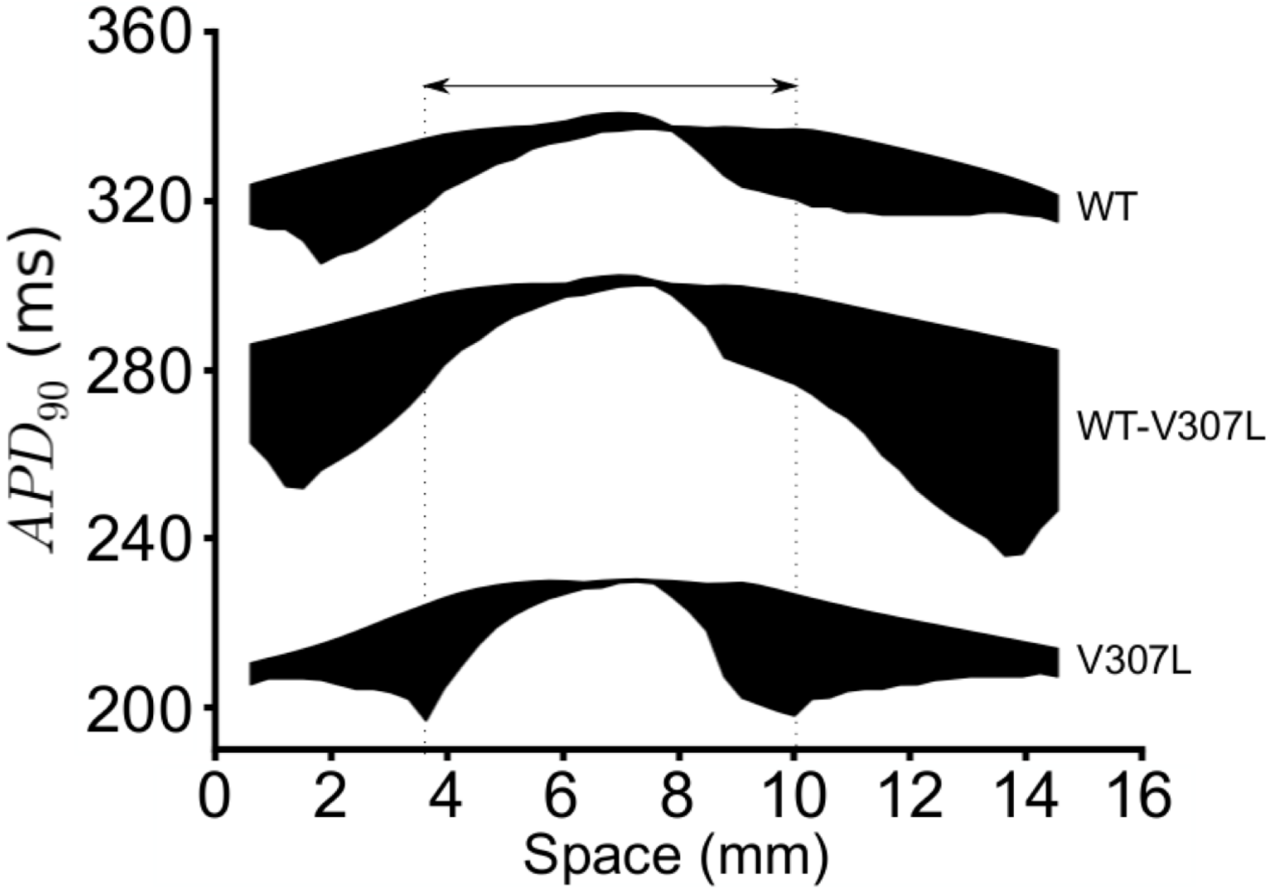

Figure S3: Vulnerable window to uni-directional conduction. Measured temporal window of vulnerability to uni-directional conduction across the transmural 1D strand in WT, WT-V307L, and V307L conditions. The percentage increase in the width of the measured vulnerable window in the region shown with an arrow compared to the WT was 37% and 82% in WT-V307L and V307L conditions, respectively.

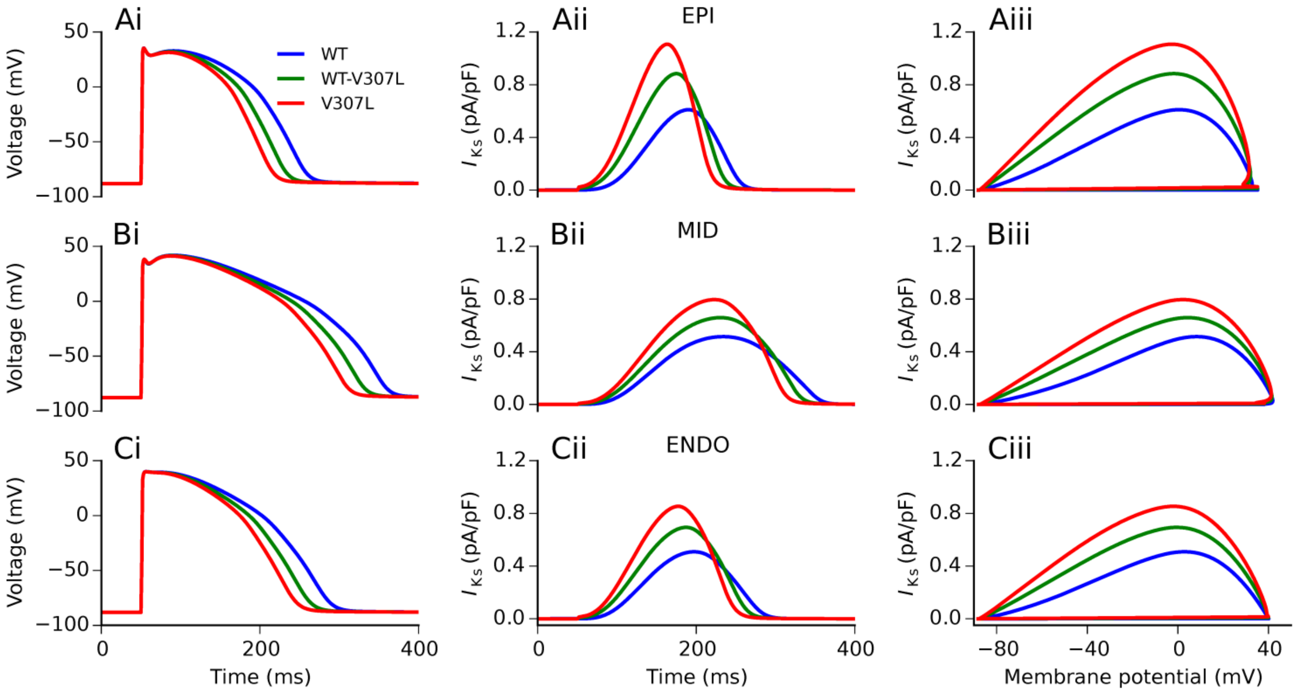

Figure S4: Action potentials and I_{Ks} profiles in the ORd model. O'Hara-Rudy dynamic (ORd) human ventricular cell action potential profiles (i), I_{Ks} profiles (ii), and corresponding I-V relationships (iii) in WT (blue), WT-V307L (green), and V307L (red) conditions for the EPI (A), MIDDLE (B), and ENDO (C) regions at a BCL of 1000 ms.

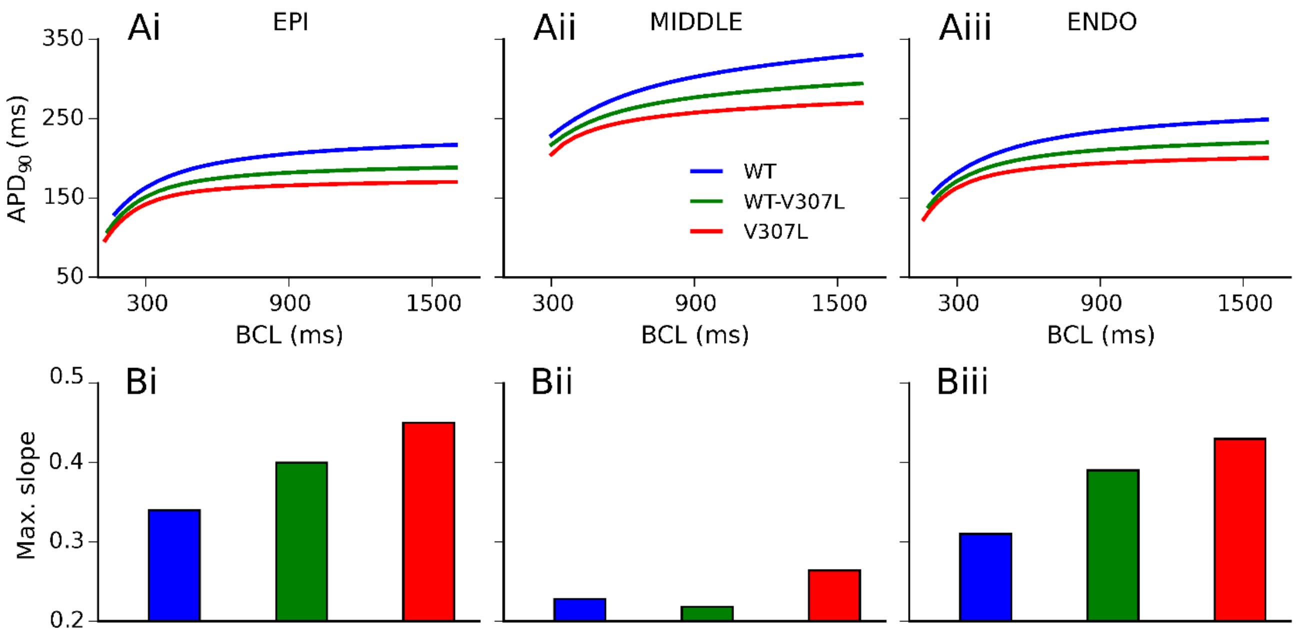

Figure S5: APD restitution curves in the ORd model. Steady state APD_{90} restitution curves in the ORd model for the EPI (i), MIDDLE (ii), and ENDO (iii) regions in WT (blue), WT-V307L (green), and V307L (red) conditions, with corresponding maximum slope of restitution (B).

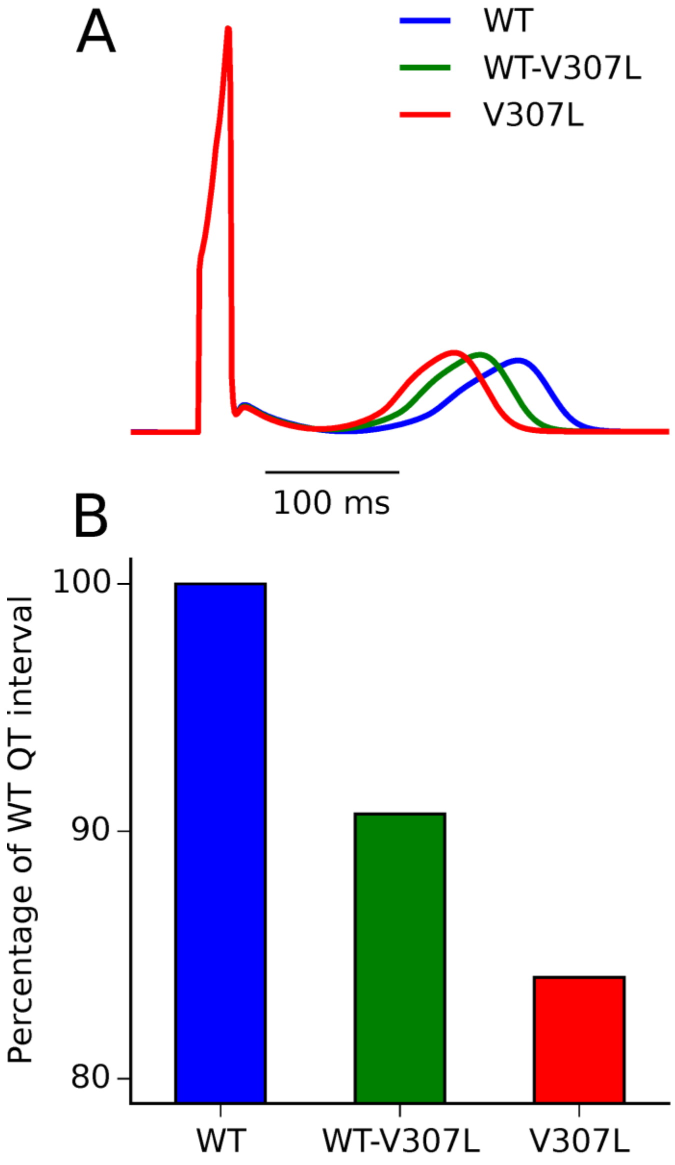

Figure S6: Pseudo-ECG measured in ORd model 1D strand. Pseudo-ECGs from the ORd model 1D transmural strand in WT, WT-V307L, and V307L conditions (A), and percentage reduction in QT interval with respect to WT (B).

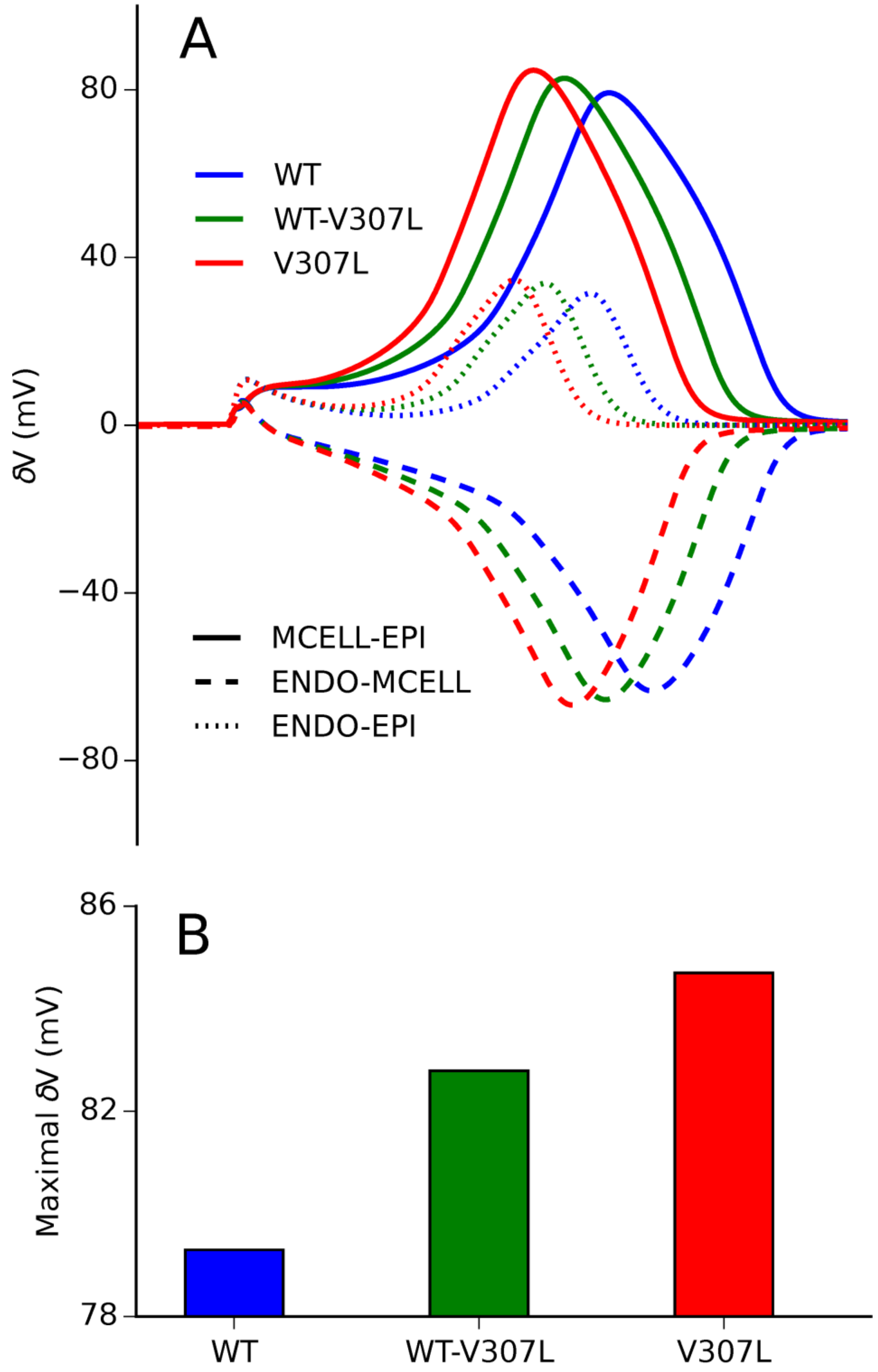

Figure S7: Membrane potential heterogeneity in ORd model. A plot of membrane potential heterogeneity (δV) in time in the ORd model (A) for WT (blue), WT-V30L (green), and V307L (red) conditions between the MIDDLE-EPI regions (solid lines), ENDO-EPI regions (dotted lines), and ENDO-MIDDLE regions (dashed lines). Maximal δV during repolarisation between MIDDLE-EPI cells (B).

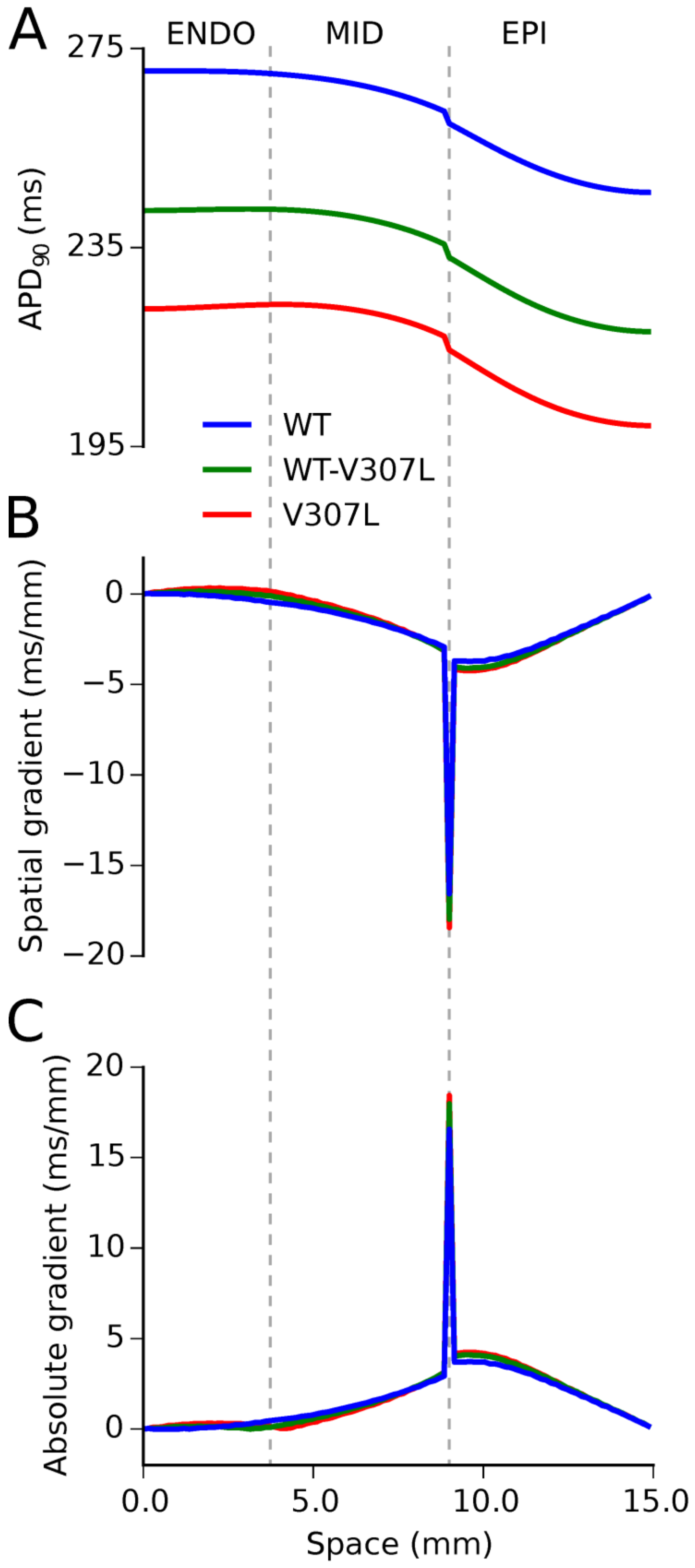

Figure S8: Spatial dispersion of APD_{90} in ORd model. Transmural dispersion of APD_{90} across the ORd model 1D strand (A) in WT (blue), WT-V307L (green), and V307L (red) conditions. Measured (B) and absolute (C) spatial gradient of APD_{90} in the 1D transmural strand.

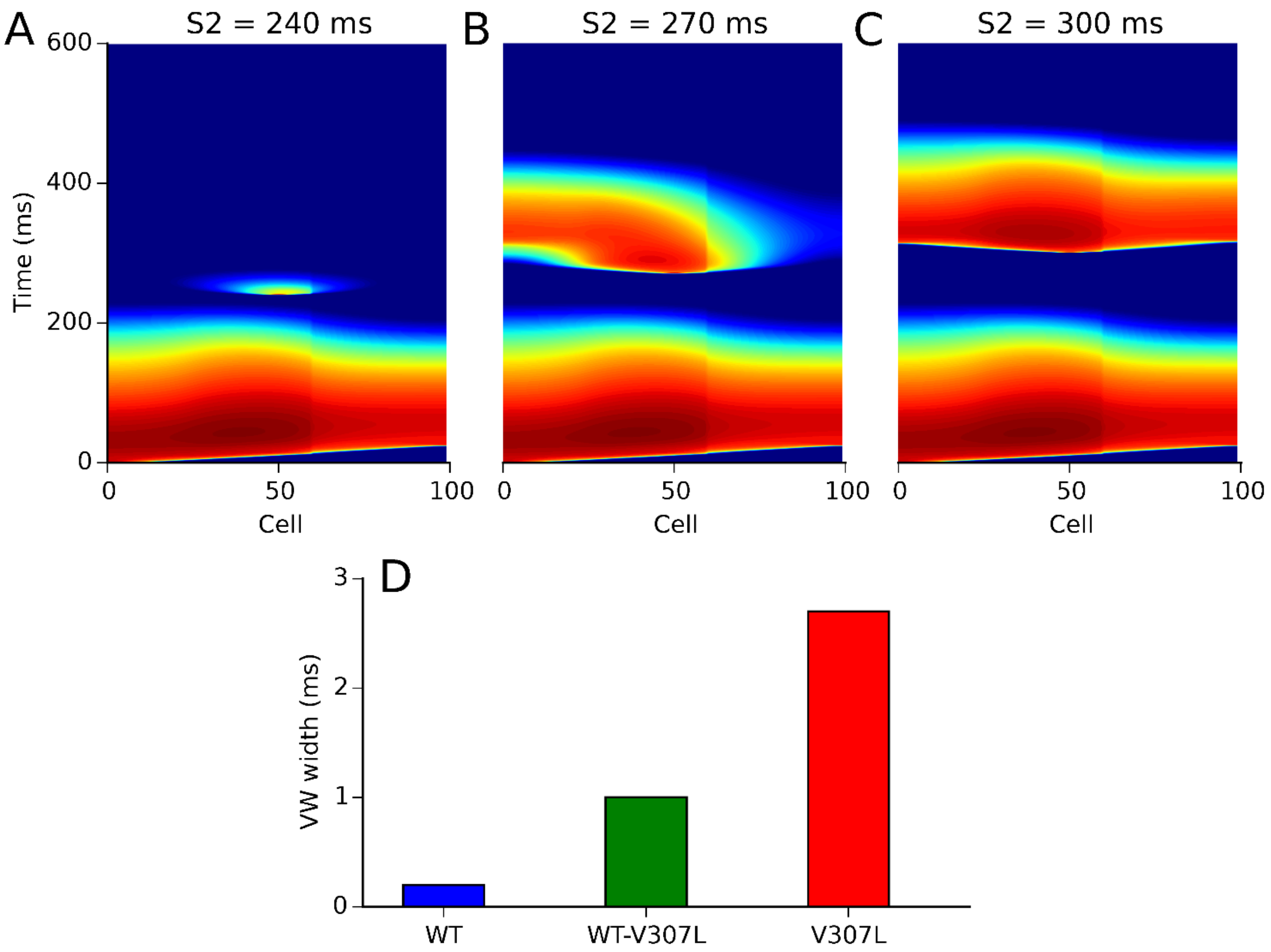

Figure S9: Vulnerable window to uni-directional conduction in ORd model. Space-time plot of AP propagation along the ORd model 1D transmural strand for application of S2 stimulus at various times, showing (A) bi-directional conduction block, (B) uni-directional conduction block, and (C) bi-directional conduction. (D) Width of temporal vulnerable window in the ORd model measured at MCELL-EPI border.

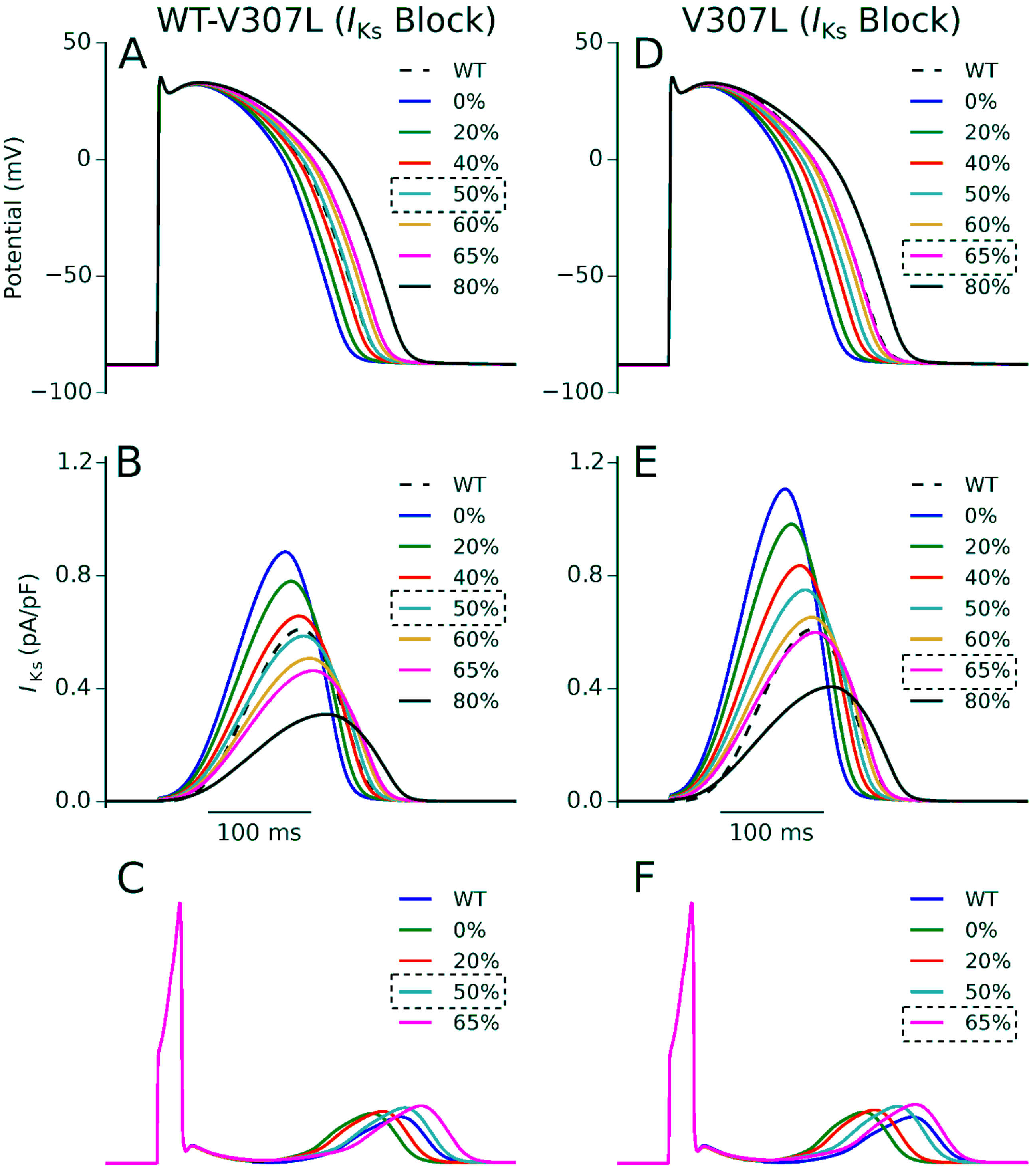

Figure S10: I_{Ks} blockade in single cell and 1D ORd model simulations. Action potentials in WT-V307L (A) and V307L (D) conditions under varying degrees of I_{Ks} blockade in the ORd model. The dashed line represents the WT and the boxed percentage represents the degree of I_{Ks} block required to normalize the APD under the respective mutation condition. I_{Ks} profiles corresponding to the ORd model APs shown in (A) and (D) are shown for WT-V307L (B) and V307L (E) conditions, respectively. Pseudo-ECGs corresponding to varying degrees of I_{Ks} blockade are shown in WT-V307L (C) and V307L (F) conditions. The blue line represents the WT and the boxed percentage represents the degree of I_{Ks} block required to normalize the QT interval under the respective mutation condition.

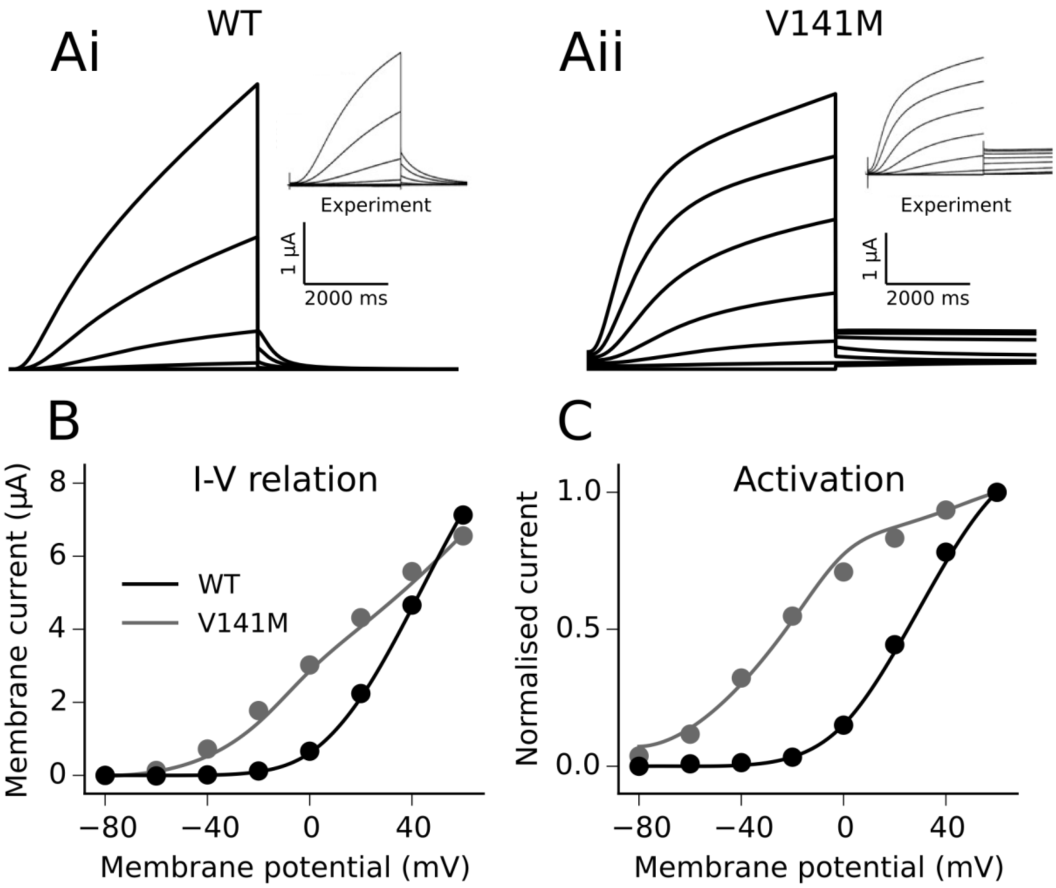

D

V141M I_{Ks} formulation

$$I_{Ks} = G_{Ks}(\xi + O_{Ks})(V_m - E_{Ks})$$

$$G_{Ks} = 0.779 \cdot \left(1 + \frac{0.6}{1 + \left(\frac{3.8 \cdot 10^{-5}}{[Ca^{2+}]_i}\right)^{1.4}}\right)$$

$$O_{Ks} = O_1 + O_2$$

$$E_{Ks} = \frac{R \cdot T}{F} \cdot \log \frac{[K^+]_o + P_{Na/K} \cdot [Na^+]_i}{[K^+]_i + P_{Na/K} \cdot [Na^+]_i}$$

$$\alpha = 2.03 \cdot 10^{-4} \cdot \exp\left\{2.60 \cdot 10^{-1} \cdot \frac{V_m \cdot F}{R \cdot T}\right\}$$

$$\beta = 2.70 \cdot 10^{-5} \cdot \exp\left\{-1.26 \cdot 10^{-1} \cdot \frac{V_m \cdot F}{R \cdot T}\right\}$$

$$\gamma = 1.25 \cdot 10^{-2} \cdot \exp\left\{5.68 \cdot 10^{-1} \cdot \frac{V_m \cdot F}{R \cdot T}\right\}$$

$$\delta = 8.59 \cdot 10^{-4} \cdot \exp\left\{-3.66 \cdot 10^{-1} \cdot \frac{V_m \cdot F}{R \cdot T}\right\}$$

$$\theta = 1.03 \cdot 10^{-2}$$

$$\eta = 2.92 \cdot 10^{-3} \cdot \exp\left\{-4.59 \cdot 10^{-1} \cdot \frac{V_m \cdot F}{R \cdot T}\right\}$$

$$\psi = 4.89 \cdot 10^{-3} \cdot \exp\left\{2.14 \cdot 10^{0} \cdot \frac{V_m \cdot F}{R \cdot T}\right\}$$

$$\omega = 5.46 \cdot 10^{-6} \cdot \exp\left\{-9.79 \cdot 10^{-1} \cdot \frac{V_m \cdot F}{R \cdot T}\right\}$$

$$\xi = 2.29 \cdot 10^{-2}$$

Figure S11: A summary of kinetics for V141M KCNQ1 mutant I_{Ks}. A comparison of simulated voltage clamp current traces in WT (Ai) and V141M (Aii) conditions using the protocol described in [35]. Simulated (solid lines) I-V relation (B) and voltage dependence of activation (C) under WT and V141M mutation conditions, compared with experimental data (points). The equations describing V141M mutant I_{Ks} are given in (D), where an additional parameter, ξ, is introduced to account for the constitutively active voltage-independent component.

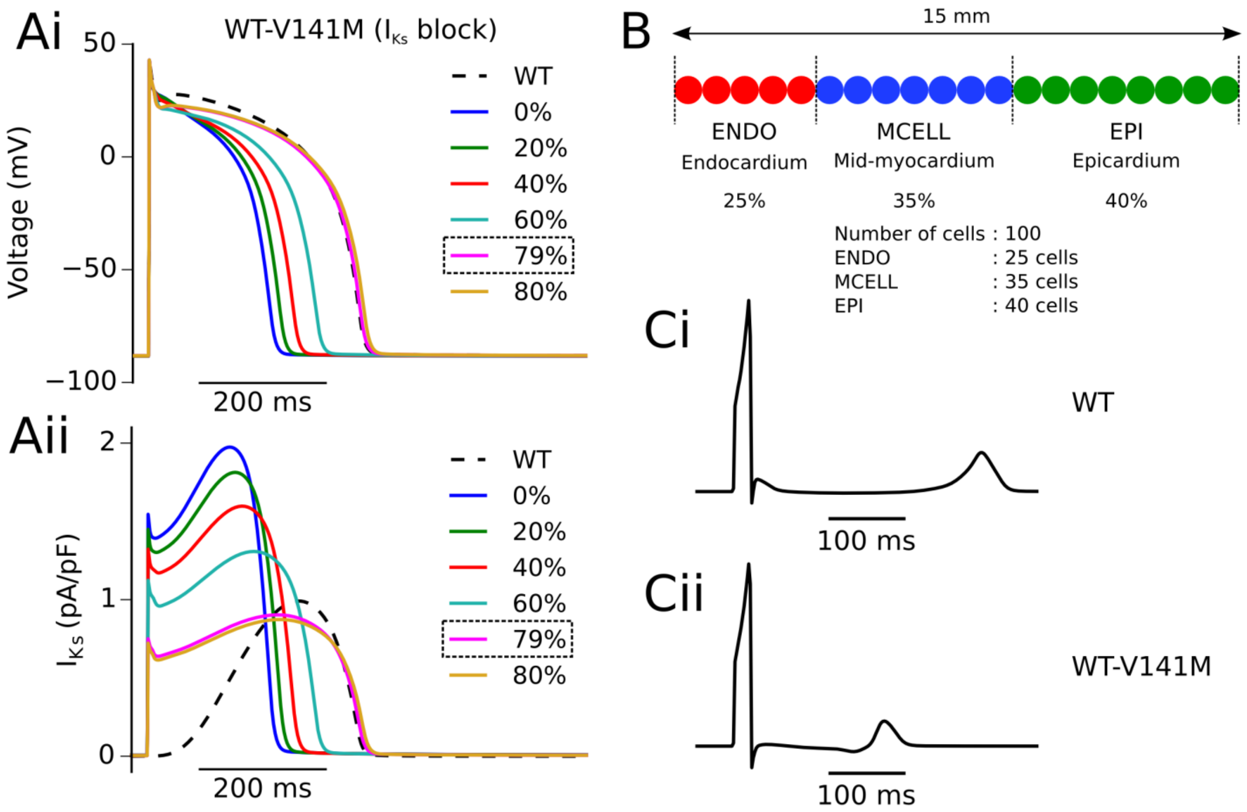

Figure S12: Single cell and 1D simulations of theV141M KCNQ1 mutation. Action potentials from endocardial cells at a pacing frequency of 1 Hz in WT-V141M conditions under varying degrees of I_{Ks} blockade (Ai). The dashed line represents the WT and the boxed percentage represents the degree of I_{Ks} block required to normalise the APD. I_{Ks} profiles corresponding to the APs shown in (Ai) are shown in (Aii). A schematic of the 1D transmural ventricular cell model is shown in (B), with corresponding pseudo ECGs measured in the WT (Ci) and WT-V141M (Cii) conditions.

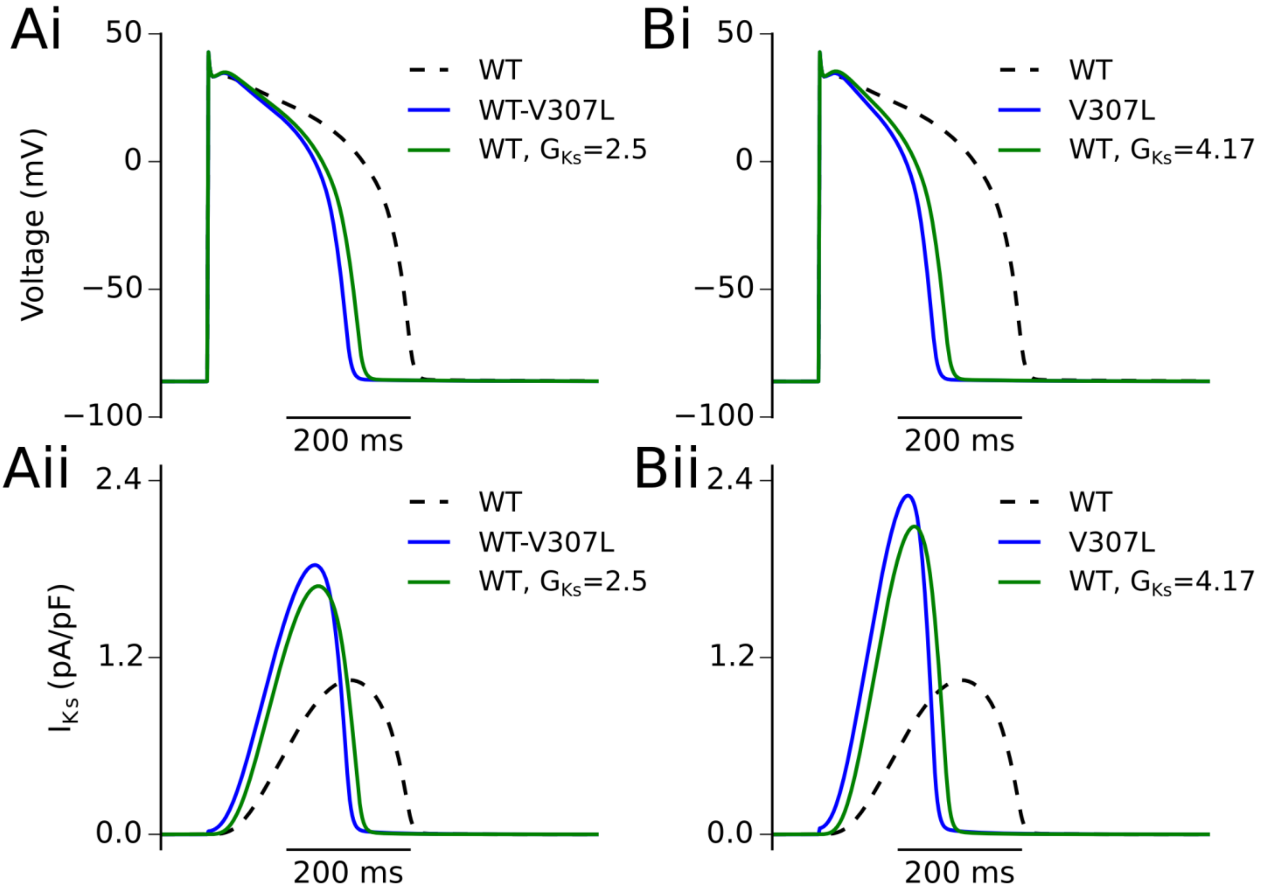

Figure S13: Effects of increasing maximal I_{Ks} conductance in the WT condition. The effect of multiplying maximal I_{Ks} channel conductance by a scaling factor, G_{Ks}, equal to the inverse of the amount of block required to normalise the AP in WT-V307L (A) and V307L (B) conditions is shown on the AP (i) and I_{Ks} profile (ii). Simulations were performed in endocardial cells at a basic cycle length of 1000 ms.

Supplementary videos

Video S1: WT reentry in 2D cross-section of ventricles. Reentry induced in the WT condition by applying a premature S2 stimulus into refractory and partially recovered tissue at 370 ms. The induced spiral wave transitions from transmural re-entry with tip rotating within the ventricle wall to brief anatomical re-entry with tip rotating around the ventricle boundary. Spiral waves self-terminate shortly after 1000 ms.

Video S2: WT-V307L reentry in 2D cross-section of ventricles. Reentry induced in the WT-V307L condition by applying a premature S2 stimulus into refractory and partially recovered tissue at 310 ms. The induced spiral wave transitions from transmural re-entry with tip rotating within the ventricle wall to anatomical re-entry with tip rotating around the ventricle boundary. Spiral waves sustain for the 5000 ms duration of the simulation.

Video S3: V307L reentry in 2D cross-section of ventricles. Reentry induced in the V307L condition by applying a premature S2 stimulus into refractory and partially recovered tissue at 230 ms. The induced transmural reentry persists and breaks up, forming regenerative multiple re-entrant wavelets. Spiral waves sustain for the 5000 ms duration of the simulation.

Video S4: WT re-entry in realistic 3D geometry (left ventricular view). Left ventricular view of re-entrant scroll waves induced in the WT condition by applying a premature S2 during the tissue refractory period at 355 ms. The initiated scroll wave quickly self-terminates in ~750 ms.

Video S5: WT re-entry in realistic 3D geometry (whole heart view). Whole heart view of re-entrant scroll waves induced in the WT condition by applying a premature S2 during the tissue refractory period at 355 ms. The initiated scroll wave quickly self-terminates in ~750 ms.

Video S6: WT-V307L re-entry in realistic 3D geometry (left ventricular view). Left ventricular view of re-entrant scroll waves induced in the WT-V307L condition by applying a premature S2 during the tissue refractory period at 315 ms. The initiated scroll wave persists and breaks up, forming regenerative multiple re-entrant wavelets which self-terminate within 2500 ms.

Video S7: WT-V307L re-entry in realistic 3D geometry (whole heart view). Whole heart view of re-entrant scroll waves induced in the WT-V307L condition by applying a premature S2 during the tissue refractory period at 315 ms. The initiated scroll wave persists and breaks up, forming regenerative multiple re-entrant wavelets which self-terminate within 2500 ms.

Video S8: V307L re-entry in realistic 3D geometry (left ventricular view). Left ventricular view of re-entrant scroll waves induced in the V307L condition by applying a premature S2 during the tissue refractory period at 260 ms. The initiated scroll wave persists and breaks up, forming regenerative multiple re-entrant wavelets. Scroll waves sustain for the 5000 ms duration of the simulation.

Video S9: V307L re-entry in realistic 3D geometry (whole heart view). Whole heart view of re-entrant scroll waves induced in the V307L condition by applying a premature S2 during the tissue refractory period at 260 ms. The initiated scroll wave persists and breaks up, forming regenerative multiple re-entrant wavelets. Scroll waves sustain for the 5000 ms duration of the simulation.

1. Shaker potassium channel gating. II: Transitions in the activation pathway. *J. Gen. Physiol.* **103,** 279–319 (1994).
2. Shaker potassium channel gating. III: Evaluation of kinetic models for activation. *J. Gen. Physiol.* **103,** 321–362 (1994).
3. Silverman, W. R., Roux, B. & Papazian, D. M. Structural basis of two-stage voltage-dependent activation in K+ channels. *Proc. Natl. Acad. Sci. U. S. A.* **100,** 2935–2940 (2003).
4. Inactivation of the sodium channel. II. Gating current experiments. *J. Gen. Physiol.* **70,** 567–590 (1977).
5. Rudy, Y. & Silva, J. R. Computational biology in the study of cardiac ion channels and cell electrophysiology. *Q. Rev. Biophys.* **39,** 57–116 (2006).
6. Nelder, J. A. & Mead, R. A Simplex Method for Function Minimization. *Comput. J.* **7,** 308–313 (1965).
7. Tusscher, K. H. W. J. ten & Panfilov, A. V. Alternans and spiral breakup in a human ventricular tissue model. *Am. J. Physiol. - Heart Circ. Physiol.* **291,** H1088–H1100 (2006).
8. Xia, L. *et al.* Simulation of Brugada syndrome using cellular and three-dimensional whole-heart modeling approaches. *Physiol. Meas.* **27,** 1125 (2006).
9. Adeniran, I., McPate, M. J., Witchel, H. J., Hancox, J. C. & Zhang, H. Increased Vulnerability of Human Ventricle to Re-entrant Excitation in hERG-linked Variant 1 Short QT Syndrome. *PLoS Comput. Biol.* **7,** (2011).
10. Adeniran, I., Harchi, A. E., Hancox, J. C. & Zhang, H. Proarrhythmia in KCNJ2-linked short QT syndrome: insights from modelling. *Cardiovasc. Res.* **94,** 66–76 (2012).
11. Clayton, R. H. *et al.* Models of cardiac tissue electrophysiology: Progress, challenges and open questions. *Prog. Biophys. Mol. Biol.* **104,** 22–48 (2011).
12. Yan, G.-X., Shimizu, W. & Antzelevitch, C. Characteristics and Distribution of M Cells in Arterially Perfused Canine Left Ventricular Wedge Preparations. *Circulation* **98,** 1921–1927 (1998).
13. Drouin, E., Charpentier, F., Gauthier, C., Laurent, K. & Le Marec, H. Electrophysiologic characteristics of cells spanning the left ventricular wall of human heart: Evidence for presence of M cells. *J. Am. Coll. Cardiol.* **26,** 185–192 (1995).
14. Zhang, H., Kharche, S., Holden, A. V. & Hancox, J. C. Repolarisation and vulnerability to re-entry in the human heart with short QT syndrome arising from KCNQ1 mutation—A simulation study. *Prog.*

Biophys. Mol. Biol. **96,** 112–131 (2008).

15. Weiss, D. L., Seemann, G., Sachse, F. B. & Dössel, O. Modelling of short QT syndrome in a heterogeneous model of the human ventricular wall. *Europace* **7,** S105–S117 (2005).
16. Gima, K. & Rudy, Y. Ionic Current Basis of Electrocardiographic Waveforms A Model Study. *Circ. Res.* **90,** 889–896 (2002).
17. Taggart, P. *et al.* Transmural repolarisation in the left ventricle in humans during normoxia and ischaemia. *Cardiovasc. Res.* **50,** 454–462 (2001).
18. Li, G.-R., Feng, J., Yue, L. & Carrier, M. Transmural heterogeneity of action potentials andI to1 in myocytes isolated from the human right ventricle. *Am. J. Physiol. - Heart Circ. Physiol.* **275,** H369–H377 (1998).
19. Glukhov, A. V. *et al.* Transmural Dispersion of Repolarization in Failing and Nonfailing Human Ventricle. *Circ. Res.* **106,** 981–991 (2010).
20. Antzelevitch, C. M Cells in the Human Heart. *Circ. Res.* **106,** 815–817 (2010).
21. Taggart, P. *et al.* Inhomogeneous Transmural Conduction During Early Ischaemia in Patients with Coronary Artery Disease. *J. Mol. Cell. Cardiol.* **32,** 621–630 (2000).
22. Seemann, G., Keller, D. U. J., Weiss, D. L. & Dossel, O. Modeling human ventricular geometry and fiber orientation based on diffusion tensor MRI. in *2006 Computers in Cardiology* 801–804 (2006).
23. Keller, D. U. J., Kalayciyan, R., Dössel, O. & Seemann, G. Fast Creation of Endocardial Stimulation Profiles for the Realistic Simulation of Body Surface ECGs. in *World Congress on Medical Physics and Biomedical Engineering, September 7 - 12, 2009, Munich, Germany* 145–148 (Springer, Berlin, Heidelberg, 2009). doi:10.1007/978-3-642-03882-2_37
24. Klepfer, R. N., Johnson, C. R. & Macleod, R. S. The effects of inhomogeneities and anisotropies on electrocardiographic fields: a 3-D finite-element study. *IEEE Trans. Biomed. Eng.* **44,** 706–719 (1997).
25. Sundnes, J., Lines, G. T. & Tveito, A. An operator splitting method for solving the bidomain equations coupled to a volume conductor model for the torso. *Math. Biosci.* **194,** 233–248 (2005).
26. Bangerth, W., Hartmann, R. & Kanschat, G. Deal.II—A General-purpose Object-oriented Finite Element Library. *ACM Trans Math Softw* **33,** (2007).
27. Finlayson, B. A. Finite element analysis: From concepts to applications. By David S. Burnett, Addision-

Wesley publishing, Reading, Ma, 844 pp., 1987. *AIChE J.* **39,** 1577–1577 (1993).

28. Durrer, D. *et al.* Total Excitation of the Isolated Human Heart. *Circulation* **41,** 899–912 (1970).
29. O'Hara, T., Virág, L., Varró, A. & Rudy, Y. Simulation of the Undiseased Human Cardiac Ventricular Action Potential: Model Formulation and Experimental Validation. *PLoS Comput. Biol.* **7,** (2011).
30. Elshrif, M. M. & Cherry, E. M. A Quantitative Comparison of the Behavior of Human Ventricular Cardiac Electrophysiology Models in Tissue. *PLoS ONE* **9,** (2014).
31. Ten Tusscher, K. H. W. J., Bernus, O., Hren, R. & Panfilov, A. V. Comparison of electrophysiological models for human ventricular cells and tissues. *Prog. Biophys. Mol. Biol.* **90,** 326–345 (2006).
32. Mann, S. A. *et al.* Convergence of models of human ventricular myocyte electrophysiology after global optimization to recapitulate clinical long QT phenotypes. *J. Mol. Cell. Cardiol.* **100,** 25–34 (2016).
33. Hancox, J. C. *et al.* In silico investigation of a KCNQ1 mutation associated with familial atrial fibrillation. *J. Electrocardiol.* **47,** 158–165 (2014).
34. Jost, N. *et al.* Contribution of I Kr and I K1 to ventricular repolarization in canine and human myocytes: is there any influence of action potential duration? *Basic Res. Cardiol.* **104,** 33–41 (2008).
35. Restier, L., Cheng, L. & Sanguinetti, M. C. Mechanisms by which atrial fibrillation-associated mutations in the S1 domain of KCNQ1 slow deactivation of IKs channels. *J. Physiol.* **586,** 4179–4191 (2008).
36. Hong, K. *et al.* De novo KCNQ1 mutation responsible for atrial fibrillation and short QT syndrome in utero. *Cardiovasc. Res.* **68,** 433–440 (2005).
37. Seebohm, G., Lerche, C., Busch, A. & Bachmann, A. Dependence of IKs biophysical properties on the expression system. *Pflüg. Arch.* **442,** 891–895 (2001).
38. O'Hara, T. & Rudy, Y. Arrhythmia formation in subclinical ('silent') long QT syndrome requires multiple insults: Quantitative mechanistic study using the KCNQ1 mutation Q357R as example. *Heart Rhythm* **9,** 275–282 (2012).
39. Moreno, J. D., Lewis, T. J. & Clancy, C. E. Parameterization for In-Silico Modeling of Ion Channel Interactions with Drugs. *PLOS ONE* **11,** e0150761 (2016).
